# Terahertz‐Wave Polarization Space‐Division Multiplexing Meta‐Devices based on Spin‐Decoupled Phase Control

**DOI:** 10.1002/advs.202412688

**Published:** 2024-12-30

**Authors:** Yuehong Xu, Yuma Takida, Tetsu Suzuki, Hiroaki Minamide

**Affiliations:** ^1^ RIKEN Center for Advanced Photonics RIKEN 519‐1399 Aramaki‐Aoba Sendai Miyagi 980‐0845 Japan

**Keywords:** metasurface, multi‐beam, polarization manipulation, space‐division multiplexing, spin‐decoupled phase control, terahertz, vector beam

## Abstract

This study presents a generalized design strategy for novel terahertz‐wave polarization space‐division multiplexing meta‐devices, functioning as multi‐polarization generators, modulators, and analyzers. It introduces the spin‐decoupled phase control method by combining gradient phase design with circular polarization multiplexing techniques, enabling exceptional flexibility in controlling the polarization directions and spatial distributions of multiple output beams. The meta‐device M‐4D is significantly demonstrated as proof of concept, which converts an incident linearly polarized wave into four beams with distinct polarization angles. Additionally, the advanced meta‐devices M‐2B and M‐4B are designed to generate two‐vector and four‐vector Bessel beams with tunable spatial polarization distributions. These meta‐devices demonstrate dynamic multi‐polarization beam modulation, validated through simulations and experiments. The proposed method significantly expands the design methodology for multi‐beam polarization control using all‐dielectric metasurfaces and holds promising potential for applications in imaging, sensing, particle manipulation, communication, and information processing. Moreover, it holds potential for adaptation to other spectral ranges.

## Graphical abstract

This study introduces spin‐decoupled phase control engineering for terahertz‐wave polarization space‐division multiplexing meta‐devices. It enables the generation of distinct polarization and vector Bessel beams, utilizing gradient grating phase design and circular polarization multiplexing. The approach enhances polarization control flexibility, with applications in terahertz imaging, communication, and processing, and potential extension across the electromagnetic spectrum.

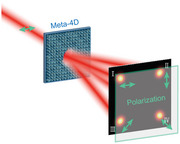

## 1 Introduction

Polarization manipulation technology has led to new scientific discoveries and breakthroughs in advanced industrial inspection techniques. It is no exception in the promising terahertz (THz)‐wave range, where polarization control and beam modulation are essential technologies to maximize the potential of nondestructive testing, sensing, and communications applications.^[^ ^1^, ^2^, ^3^, ^4^ ^]^ As data communication volumes grow exponentially, polarization and space‐division multiplexing technologies can significantly increase the number of channels and expand information capacity to meet the growing transmission demands.^[^ ^5^, ^6^, ^7^ ^]^ Meanwhile, the application of multi‐polarization/Stokes imaging,^[^ ^8^, ^9^, ^10^ ^]^ vector beams,^[^ ^11^, ^12^ ^]^ and Bessel beams can improve lateral resolution and penetration depth,^[^ ^13^ ^]^ further enhancing imaging quality and sensing accuracy. Researching and implementing THz‐wave polarization space‐division multiplexing (TPSM) devices is essential for improving the performance and effectiveness of THz technology, thereby fully realizing its potential.

A metasurface is composed of subwavelength structures arranged in a 2D array, making it a thin, flat, and multifunctional element.^[^ ^14^ ^]^ Beyond its capability to manipulate amplitude and phase,^[^ ^15^, ^16^ ^]^ one of its most notable features is its ability to achieve diverse types of polarization conversion and separation through precise control of electromagnetic waves.^[^ ^17^, ^18^, ^19^, ^20^, ^21^, ^22^ ^]^ This enables the creation of efficient and compact TPSM meta‐devices.^[^ ^23^, ^24^ ^]^ To get these meta‐devices, there are two main approaches according to polarization‐phase design principles. One approach uses dynamic phase control, which enables the dynamic phase adjustment of two orthogonal eigen linearly polarized (LP) waves by altering the size and shape of the unit cell structures.^[^ ^24^, ^25^, ^26^, ^27^ ^]^ Another approach utilizes geometric phase (also called Pancharatnam–Berry phase, PB phase) control,^[^ ^28^ ^]^ which enables positive or negative identical phase changes of right‐handed circularly polarized (RCP) and left‐handed circularly polarized (LCP) waves by rotating identical unit cell structures.^[^ ^29^, ^30^, ^31^, ^32^, ^33^, ^34^ ^]^ In the case of a THz‐wave four‐beam steering device designed using the aforementioned approaches, it can only generates four beams with either identical polarization states,^[^ ^27^, ^29^, ^32^, ^33^, ^34^ ^]^ or just two types,^[^ ^26^, ^32^ ^]^ but cannot produce four beams with distinct polarization angles. The spin‐decoupled phase control has the potential to effectively overcome the aforementioned limitations by introducing both dynamic and geometric phases within a single unit cell structure, enabling more complex and rich functionalities.^[^ ^22^, ^35^, ^36^, ^37^, ^38^ ^]^ Meanwhile, all‐dielectric metasurfaces outperform metallic ones in achieving full phase control (0–2π) and higher transmission efficiency in single‐layer transmission‐type designs.^[^ ^39^ ^]^ Silicon‐based metasurfaces are also compatible with established semiconductor integrated photonics, making all‐dielectric high‐resistivity (HR) silicon ideal for designing and fabricating THz‐wave meta‐devices.

In this work, inspired by spin‐decoupled phase control and based on HR silicon, we propose a design method for versatile TPSM devices combing circular polarization multiplexing technology. A THz‐wave four‐beam steering meta‐device that converts an incident LP wave into four beams with distinct polarization angles is designed and experimentally characterized to demonstrate the effectiveness of the proposed method. Here, we denote it as M‐4D. This proposed method also shows the capability to design versatile four‐beam polarization converters that can produce beams with either identical or different polarization states. To expand this approach, a two‐vector Bessel beam generator, referred to as M‐2B, and a four‐vector Bessel beam generator, referred to as M‐4B, were designed. These designs have positive effects on achieving greater imaging depth, enhanced lateral resolution, active control in particle manipulation and high‐capacity information transmission. The proposed advanced TPSM meta‐devices broaden and enhance the applications of metasurfaces, making them suitable for specific polarization‐related systems such as THz‐wave sensing, imaging, communication, and information processing.

## 2 Results

### 2.1 Meta‐Device M‐4D for Generating Four Distinct Polarized Beams

**Figure 1:**
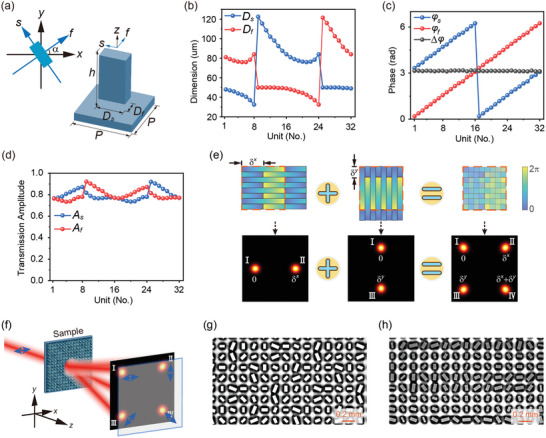
Units and methodology for designing TPSM meta‐devices. a) Schematic of the basic unit cell composed of a HR silicon pillar. b) Lateral dimensions of the 32 silicon pillars. c) Eigen phase shift profiles of *φ_f_* and *φ_s_* and their phase difference Δ*φ* = *φ_s_* – *φ_f_* at 1.0 THz. d) Eigen transmission amplitude profiles of *A_f_* and *A_s_* of the 32 units at 1.0 THz. e) Phase design schematic of a four‐beam generator with initial phase differences. f) Functional demonstration diagram of M‐4D. g,h) Micrographs of the fabricated M‐4D and M‐2B, respectively.

Considering a half‐wave plate unit structure with its fast and slow axes oriented at an angle *α* relative to the global *x*‐axis, as illustrated in **Figure** **1**a, the transmitted wave's Jones vector for an incident circularly polarized (CP) wave expressed by ${\left[1\;\;\;i\sigma \right]}^{T}/\sqrt{2}$, where σ = +1 represents the LCP wave and σ = −1 represents the RCP wave, is given by:

(1) $$\begin{array}{c} E_{\mathrm{out}}=M\left(-\alpha \right)\times J\times M\left(\alpha \right)\times \frac{1}{\sqrt{2}}\left[\begin{array}{c} 1\\ i\sigma \end{array}\right]=e^{i\left(\varphi_{f}+2\alpha \sigma \right)}\times \frac{1}{\sqrt{2}}\left[\begin{array}{c} 1\\ -i\sigma \end{array}\right] \end{array}$$

where ***M*** (*α*) = [cos(*α*) sin(*α*); −sin(*α*) cos(*α*)] is the rotation matrix of the unit structure and ***J*** = $e^{i\varphi_{f}}$[1 0; 0 −1] is the Jones matrix of the half‐wave plate with introducing an additional dynamic phase *φ_f_* representing the initial phase along the fast axis. This equation shows that the transmitted cross‐LCP wave carries a phase *φ_lr_* = *φ_f_* −2*α* under RCP incidence; the transmitted cross‐RCP wave carries a phase *φ_rl_* = *φ_f_* + 2*α* under LCP incidence. This phase control method overcomes the spin‐locked limitation of the PB phase control method, where cross‐LCP and cross‐RCP waves always carry phases of equal magnitude but opposite signs (±2*α*). It allows for independent and free control of the phases of the cross‐LCP and cross‐RCP waves by varying *φ_f_* from 0 to 2π, enhancing the design flexibility of metasurfaces. This approach is referred to as spin‐decoupled phase control.^[^ ^36^, ^37^ ^]^ For an all‐dielectric sub‐wavelength structure, varying its size adjusts the eigen phase shift of *φ_f_* and *φ_s_*. Based on the database at 1.0 THz in previous work,^[^ ^25^ ^]^ 32 HR silicon pillars were selected (see Section S1, Supporting Information for selection criteria). Figure 1a shows the schematic of the unit cell with a period *P* = 150 µm, height *h* = 200 µm, and dimensions *D_f_* and *D_s_* along the fast and slow axes, respectively. Their corresponding lateral dimensions are listed in Figure 1b. It should be noted that the substrate serves to support the pillars, with both the substrate and pillars set as lossless silicon with a relative permittivity of 11.9. The eigen phase shift profiles of *φ_f_* and *φ_s_* and their phase difference Δ*φ* = *φ_s_* – *φ_f_* are shown in Figure 1c. The phase response of the 32 structures covers the full dynamic range from 0 to 2π, with Δ*φ* close to π. Figure 1d shows the eigen transmission amplitude profiles, which represent the magnitude of the transmission coefficient, for *A_f_* and *A_s_* along the fast and slow axes. Both *A_f_* and *A_s_* exhibit high transmission amplitude with average values of 0.8 and minimal difference. The phase and transmission responses indicate that the selected 32 structures function as quasi‐half‐wave plates. Therefore, under CP incidence, the transmission amplitudes of both cross‐RCP and cross‐LCP waves remain high and equal. Thanks to the eigen dynamic phase *φ_f_*, the phases of transmitted cross‐CP waves also cover the full dynamic range from 0 to 2π (see Section S2, Supporting Information for detailed CP response). Based on the 32 structures, the transmitted cross‐LCP and cross‐RCP phases with spin‐decoupled control can be design independently, enabling more flexible TPSM meta‐devices.

According to the generalized Snell's law,^[^ ^14^ ^]^ by configuring the phase in transverse *x*‐direction to have equal but opposite grating phase gradients for odd and even rows, with an initial phase difference *δ_x_*, Beams I and II are symmetrically deflected. Their respective phases are *ξ* ^I^ = 0 and *ξ* ^II^ = *δ_x_*, as illustrated in Figure 1e. Similarly, in the longitudinal *y*‐direction, the phase design for odd and even columns with an initial phase difference *δ_y_* results in the generation of Beams I and III, with phases *ξ* ^I^ = 0 and *ξ* ^III^ = *δ_y_*, respectively. By setting the absolute phase gradients equal in both directions and combining the transverse and longitudinal phase distributions, four beams (I, II, III, IV) are symmetrically deflected around the normal. The corresponding phases are *ξ* ^I^ = 0, *ξ* ^II^ = *δ_x_*, *ξ* ^III^ = *δ_y_*, and *ξ* ^IV^ = *δ_x_* + *δ_y_*. RCP and LCP waves with identical amplitudes and phases *ξ_r_* and *ξ_l_* combines to form a LP wave with an orientation angle *γ* = (*ξ_r_* – *ξ_l_*)/2 = Δ*ξ*/2. Therefore, the LP output orientation angle can be controlled by adjusting the phase difference Δ*ξ* of the output LCP and RCP components. By employing circular polarization complexing technique in polarization space‐division multiplexing meta‐devices, the polarization orientation angle of each beam can be precisely controlled by adjusting the phase difference Δ*ξ^Ω^* between each beam's LCP and RCP components (where *Ω* ∈ {I, II, III, IV} represents the beam identifier).

The spin‐decoupled phase control enables the metasurface to be designed freely, ensuring that the modulated transmitted cross‐LCP and cross‐RCP phase distributions meet below specific conditions:

(2) $$\Psi_{lr}^{M-4D}=\Phi_{f}-2A=\Psi_{lr}^{x}+\Psi_{lr}^{y}$$

(3) $$\Psi_{rl}^{M-4D}=\Phi_{f}+2A=\Psi_{rl}^{x}+\Psi_{rl}^{y}$$

Here, *Φ_f_* and *A* represent the dynamic phase distribution and rotation angle distribution, respectively. *Ψ_pq_^x^* and *Ψ_pq_^y^* denote the periodic phase gradient distributions in the transverse and longitudinal directions, respectively, with *pq* ∈ {*rl*, *lr*}, where *p* (*q*) indicates the handedness of the output (input) CP wave. The corresponding equations are as follows:

(4) $$\Psi_{lr}^{x}=\left\{\begin{array}{c} 2\pi n_{x}/N,\left(n_{x}=1,3,5,\text{…},2N_{x}-1\right)\\ -2\pi n_{x}/N+\delta_{lr}^{x},\left(n_{x}=2,4,6,\text{…},2N_{x}\right) \end{array}\right.$$

(5) $$\Psi_{lr}^{y}=\left\{\begin{array}{c} 2\pi n_{y}/N,\left(n_{y}=1,3,5,\text{…},2N_{y}-1\right)\\ -2\pi n_{y}/N+\delta_{lr}^{y},\left(n_{y}=2,4,6,\text{…},2N_{y}\right) \end{array}\right.$$

(6) $$\Psi_{rl}^{x}=\left\{\begin{array}{c} 2\pi n_{x}/N+\delta_{0},\left(n_{x}=1,3,5,\text{…},2N_{x}-1\right)\\ -2\pi n_{x}/N+\delta_{0}+\delta_{rl}^{x},\left(n_{x}=2,4,6,\text{…},2N_{x}\right) \end{array}\right.$$

(7) $$\Psi_{rl}^{y}=\left\{\begin{array}{c} 2\pi n_{y}/N+\delta_{0},\left(n_{y}=1,3,5,\text{…},2N_{y}-1\right)\\ -2\pi n_{y}/N+\delta_{0}+\delta_{rl}^{y},\left(n_{y}=2,4,6,\text{…},2N_{y}\right) \end{array}\right.$$

Here, *N* is the order of the phase gradient; (*n_x_P*, *n_y_P*) are the position coordinates, where *P* is the period of the unit cell, *n_x_* ∈ [1, 2*N_x_*], *n_y_* ∈ [1, 2*N_y_*]; 2*N_x_* × 2*N_y_* represents the number of units in the entire metasurface, where *N_x_* and *N_y_* are positive integers; *δ*_0_ is the designed intrinsic phase difference between cross‐RCP and cross‐LCP; *δ_pq_^x^* and *δ_pq_^y^* represent the initial phase differences in the transverse and longitudinal directions, respectively. Under RCP incidence, the four transmitted cross‐LCP beams carry phases *ξ_lr_* ^I^ = 0, *ξ_lr_* ^II^ = *δ_lr_^x^*, *ξ_lr_* ^III^ = *δ_lr_^y^*, and *ξ_lr_* ^IV^ = *δ_lr_^x^* + *δ_lr_^y^*, separately. Under LCP incidence, the four transmitted cross‐RCP beams carry phases *ξ_rl_^I^ = δ*_0_, *ξ_rl_* ^II^ = *δ*_0_ + *δ_rl_^x^*, *ξ_lr_* ^III^ = *δ*_0_ + *δ_rl_^y^*, and *ξ_lr_* ^IV^ = *δ*_0_ + *δ_rl_^x^* + *δ_rl_^y^*, separately. For one incident LP wave with an orientation angle *γ* ^in^, it decomposes into RCP and LCP components with equal amplitudes and phases *ξ_r_* ^in^ = *γ* ^in^ and *ξ_l_* ^in^ = −*γ* ^in^. Therefore, assuming equal conversion efficiencies of the deflected cross‐LCP and cross‐RCP waves, the transmitted beams will remain LP with orientation angles given by:

(8) $$\gamma^{\Omega }=-\gamma^{\mathrm{in}}+\left(\xi_{rl}^{\Omega }-\xi_{lr}^{\Omega }\right)/2$$

Consequently, the orientation angles of the four LP waves are expressed as:

(9) $$\gamma^{I}=-\gamma^{\mathrm{in}}+\delta_{0}/2$$

(10) $$\gamma^{\mathrm{II}}=\gamma^{I}+\left(\delta_{rl}^{x}-\delta_{lr}^{x}\right)/2$$

(11) $$\gamma^{\mathrm{III}}=\gamma^{I}+\left(\delta_{rl}^{y}-\delta_{lr}^{y}\right)/2$$

(12) $$\gamma^{\mathrm{IV}}=\gamma^{I}+\left(\delta_{rl}^{x}-\delta_{lr}^{x}\right)/2+\left(\delta_{rl}^{y}-\delta_{lr}^{y}\right)/2$$

In this work, *δ*_0_ = *δ_lr_^x^* = *δ_lr_^y^* = 0, *δ_rl_^x^* = π, *δ_rl_^y^* = π/2, *N* = 8 and 2*N_x_* = 2*N_y_* = 120 are set to demonstrate a TPSM meta‐device, referred to as M‐4D. Under LP incident waves with orientation angles *γ* ^in^, the polarization orientation angles of the four deflected LP waves are *γ* ^I^ = −*γ* ^in^, *γ* ^II^ = −*γ* ^in^ + π/2, *γ* ^III^ = −*γ* ^in^ + π/4, and *γ* ^IV^ = −*γ* ^in^ + 3π/4, respectively. Both the beam deflection angles in *x‐* and *y*‐directions have absolute values of sin^−1^[*λ*/(*NP*)] = 14.5°, where *λ* = 300 µm is the wavelength at 1.0 THz. Figure 1f illustrates its functionality. Appropriate units from the design library were selected and arranged according to Equations (2) and (3).

**Figure 2:**
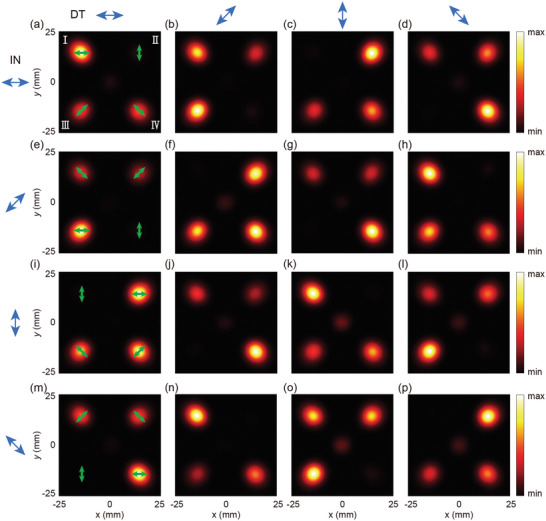
Theoretical prediction of the polarization direction and corresponding and simulation results of M‐4D. (a–d), (e–h), (i–l), and (m–p) Electric field intensity components at 0, π/4, π/2, and 3π/4 polarization directions under LP incidence with *γ* ^in^ = 0, π/4, π/2, and 3π/4, respectively, where the green arrows indicate theoretically predicted polarization direction of each beam. The blue arrows in the left column represent the incident polarization (IN), while those in the top row represent the detection polarization (DT).

Ansys HFSS was used for electromagnetic field simulation of M‐4D to characterize the output beam patterns. Due to memory constraints, the modeled M‐4D structure was limited to a 36×36 unit array with a substrate thickness of 100 µm. The excitation source was set to a 1.0 THz Gaussian LP wave with a 1 mm radius for four cases: *γ* ^in^ = 0, π/4, π/2, and 3π/4. And the excitation source port is positioned on the underside of the substrate to eliminate any influence of substrate thickness on the simulation results. Perfect absorbers were set around the perimeter to prevent interference from boundary reflections. The electric fields at *z* = 55 mm from the metasurface were calculated from those at 0.5 mm using Huygens‐Fresnel theory, with the results presented in **Figure** **2**. Under LP incidence with an orientation angle of *γ* ^in^ = 0, the electric field intensity components of LP outputs with orientation angles of 0, π/4, π/2, and 3π/4 were extracted, as shown in Figure 2a–d. Each intensity map shows a missing beam, indicating that its polarization is perpendicular to the detected polarization direction. Consequently, the orientation angles of the four output LP beams are *γ* ^I^ = 0, *γ* ^II^ = π/2, *γ* ^III^ = π/4, and *γ* ^IV^ = 3π/4, respectively, aligning with the theoretical prediction indicated by the green arrows in Figure 2a. For other incident orientation angles, results are presented in Figure 2e–p, showing changes in the polarization direction of each beam. For instance, Beam‐I's LP orientation angle is 0, 3π/4, π/2 and π/4 for *γ* ^in^ = 0, π/4, π/2, and 3π/4, respectively. On the observation plane at *z* = 55 mm, the spacing between the four deflected beams in both the *x‐* and *y‐*directions is 28.4 mm, matching the theoretical value 2*z*⋅tan(14.5°) = 28.4 mm. All results align with the design. Additionally, data from the center points of each beam were extracted to analyze the corresponding polarization ellipses, further confirming the above analysis (see Section S4, Supporting Information for details). Notably, regardless of the incident LP orientation angle, the polarization directions of the output LP beams keep specific relationships: Beams I and II are always perpendicular to each other, as are Beams III and IV. Additionally, the orientation angle of Beam III is consistently π/4 relative to Beam I. The intensity of the four beams can be controlled by integrating polarizers that positioned before and after M‐4D. Rotating a polarizer on either side allows adjustment of the intensity of the four output beams. Fixing one polarizer and rotating the other enables switching a specific beam on or off. These characteristics of M‐4D have significant applications in active control systems. Under four incident polarizations, the simulated average total conversion efficiency of the four beams is ≈22.72%. It should be noted that, based on the proposed phase design method, the existence of high‐order diffraction waves propagating to the far field and evanescent waves localized in the near field imposes a limitation on the efficiency of the four working beams. The theoretical maximum efficiency achievable with this phase design method is 25%. The appropriate adjustment of *N* and *P* parameters effectively suppresses stray high‐order diffraction orders, leading to purer working beams in the far field (see Section S12, Supporting Information for details).

**Figure 3:**
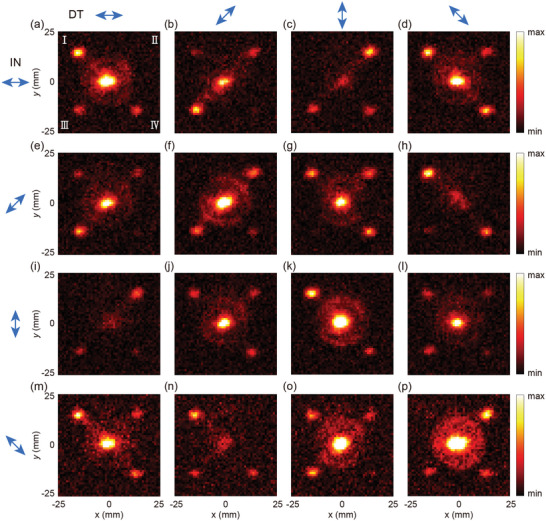
Experimental results of M‐4D. (a–d), (e–h), (i–l), and (m–p) Electric field intensity components at 0, π/4, π/2, and 3π/4 polarization directions under LP incidence with *γ* ^in^ = 0, π/4, π/2, and 3π/4, respectively. The blue arrows in the left column represent the incident polarization (IN), while those in the top row represent the detection polarization (DT).

The M‐4D was fabricated using conventional lithography combined with deep reactive ion etching on a HR silicon wafer. The wafer is a 1‐mm‐thick, undoped, intrinsic silicon substrate with a resistivity exceeding 20 kΩ·cm, exhibiting negligible absorption (see Section S3, Supporting Information for the measured dielectric parameters of the HR silicon wafer). Both surfaces of the wafer are polished to achieve a high‐quality finish. Figure 1g shows the microscope image of the center part of the fabricated sample. The performance of the M‐4D was evaluated using an experimental setup consisting of an injection‐seeded THz‐wave parametric generator and a THz‐wave detector.^[^ ^40^ ^]^ **Figure** **3** presents the experimental results, showing the electric field intensity components for detected polarization directions of 0, π/4, π/2, and 3π/4 under LP incidence with corresponding *γ* ^in^ values of 0, π/4, π/2, and 3π/4. The measured patterns closely match the simulated ones shown in Figure 2, validating the theoretical design's effectiveness and practicality for real‐world applications. The measured spacing between the four deflected beams in both *x‐* and *y‐*directions is 28 mm, which is nearly consistent with the theoretical and simulated values. The slight deviation is attributed to factors such as the scanning step size, resolution, and errors in the measurement of the *z*‐distance. It is noted that the center beam observed in the measured patterns is caused by the direct transmission of the incident beam through the sample. Under four different incident polarization states, the experimental results show that the average total conversion efficiency of the four beams is ≈16.46%. The slightly lower performance observed in the experimental results compared to the simulations may be attributed to various factors. First, a significant factor may be the detrimental effect of Fabry–Pérot (FP) interference arising from the 1 mm‐thick wafer. Multiple complex reflections between the metasurface and the substrate's bottom surface ultimately degrades performance. This issue can be addressed by applying an anti‐reflective coating to the substrate's bottom surface to eliminate FP interference. And it can be mitigated by employing a substrate material with a lower refractive index to reduce the interference. In the time‐domain system, FP interference can be eliminated by truncating the delayed reflected signal resulting from the thick substrate (see Section S10, Supporting Information for details). Additionally, fabrication inconsistencies, including variations in size, uniformity, and material refractive index, likely impacted the basic unit's half‐wave plate performance, reducing the overall efficiency and functionality of the device.^[^ ^38^ ^]^ The measured silicon pillars exhibit a lateral dimension reduced to ≈95% of the designed value, a height of ≈190 µm, a relative permittivity of 11.67, and a dielectric loss tangent of 0.0002. Simulations conducted under these parameters reveal a significant zeroth‐order direct transmission beam, with the efficiency reduced to 18.91% (see Section S11, Supporting Information for details). Furthermore, experimental results may be affected by measurement errors, including detector misalignment (e.g., angular inaccuracies) and system instability.

The proposed method leverages spin‐decoupled phase control, gradient phase design, and circular polarization multiplexing to facilitate the creation of TPSM devices, such as the M‐4D meta‐device, as well as other versatile meta‐device configurations. For example, by setting *δ_rl_^x^* = π and *δ_rl_^y^* = π, the orientation angles of the four LP beams under LP incidence with *γ* ^in^ are *γ* ^I^ = *γ* ^IV^ = −*γ* ^in^, *γ* ^II^ = *γ* ^III^ = −*γ* ^in^ + π/2. This means that Beam‐I and Beam‐IV have the same polarization direction, as do Beam‐II and Beam‐III, with the polarization directions of Beam‐I/Beam‐IV being perpendicular to those of Beam‐II/Beam‐III. Similarly, by setting *δ_rl_^x^* = π/2 and *δ_rl_^y^* = π/2, the output orientation angles become *γ* ^I^ = −*γ* ^in^, *γ* ^II^ = *γ* ^III^ = −*γ* ^in^ + π/4, and *γ* ^IV^ = −*γ* ^in^ + π/2, indicating that Beam‐II and Beam‐III share the same polarization direction, while Beam‐I and Beam‐IV are perpendicular to each other. By setting *δ_rl_^x^* = π/3 and *δ_rl_^y^* = 2π/3, the output orientation angles become *γ* ^I^ = −*γ* ^in^, *γ* ^II^ = −*γ* ^in^ + π/6, *γ* ^III^ = −*γ* ^in^ + π/3, and *γ* ^IV^ = −*γ* ^in^ + π/2, with each angle differing sequentially by π/6. Compared to previous research, where output beams typically exhibited identical or spatially symmetric polarization states, the proposed method offers significantly greater flexibility in polarization control. It enables the design of four‐beam polarization devices with polarization orientations that can be identical, distinct, symmetric, or asymmetric, depending on the desired configuration. The proposed meta‐devices demonstrate significant potential for applications in THz‐wave polarization imaging when integrated with four THz‐wave detector units or a THz‐wave detector array. By enabling the simultaneous generation of beams with distinct polarization states, these devices can substantially reduce imaging time by eliminating the need for mechanical modulation through rotating polarizers. Furthermore, they simplify the system architecture and reduce the overall physical footprint. The devices’ thin and compact design, coupled with their high functionality, allows for the production of multiple polarization orientations without the need for complex configurations or bulky optical components, offering an efficient solution for advanced THz systems.

### 2.2 Meta‐Devices for Generating Diverse Vector Bessel Beams

As an important extension of the proposed method, this approach enables the design of more versatile THz polarization space‐division multiplexing (TPSM) devices, such as cylindrical vector beams and Bessel beams, by integrating specific beam phase profiles. To demonstrate the feasibility of this concept, we developed the meta‐device M‐2B, which generates two vector Bessel beams, each with distinct spatial polarization distributions. Under CP incidence, the modulated cross‐RCP and cross‐LCP phases by M‐2B are designed as follows:

(13) $$\begin{array}{c} \Psi_{lr}^{M-2B}=-2\pi r/U+m_{l}\theta +\left\{\begin{array}{c} 2\pi n_{y}/N,\left(n_{y}=1,3,5,\text{…},2N_{y}-1\right)\\ -2\pi n_{y}/N+\delta_{lr}^{y},\left(n_{y}=2,4,6,\text{…},2N_{y}\right) \end{array}\right. \end{array}$$

(14) $$\begin{array}{ccc} \Psi_{rl}^{M-2B} & = & -2\pi r/U+m_{r}\theta \\ & & +\left\{\begin{array}{c} 2\pi n_{y}/N+\delta_{0},\left(n_{y}=1,3,5,\text{…},2N_{y}-1\right)\\ -2\pi n_{y}/N+\delta_{0}+\delta_{rl}^{y},\left(n_{y}=2,4,6,\text{…},2N_{y}\right) \end{array}\right. \end{array}$$

The last term in the equation represents the phase distribution for generating two deflected beams in the longitudinal direction, while the first two terms correspond to the Bessel phase distribution.^[^ ^38^, ^41^ ^]^ Here, (*r*, *θ*) are position coordinates in the polar coordinate system, corresponding to (*n_x_P−N_x_P*, *n_y_P−N_y_P*) in the Cartesian coordinate system. *m_r_* and *m_l_* refer to the topological charges, which represent the orbital angular momentum (OAM) carried by the RCP and LCP waves, respectively. *U* denotes the radial period, encompassing a 2π phase. For LP incident wave with an orientation angle of *γ* ^in^, the electric field of the transmitted Beam‐Ω is express as

(15) $$E_{\mathrm{out}}^{\Omega }\propto e^{i\left(-2\pi r/U\right)}\left[\begin{array}{c} \cos\left(\frac{m_{r}-m_{l}}{2}\theta +\gamma^{\Omega }\right)\\ \sin\left(\frac{m_{r}-m_{l}}{2}\theta +\gamma^{\Omega }\right) \end{array}\right]$$

The phase term −2π*r*/*U* indicates that both transmitted beams are Bessel beams carrying OAM with *m* = 0. The Jones vector term reveals that the localized polarization orientation is given by [(*m_r_* − *m_l_*)/2]*θ* + *γ^Ω^*, where *γ^Ω^* represents the polarization rotation caused by the incident LP wave and the gradient grating phase, as described in Equation (8). As a simple example to demonstrate M‐2B, the following parameters are set: *δ*_0_ = *δ_lr_^y^* = 0, *δ_rl_^y^* = π, *N* = 8, 2*N_x_* = 2*N_y_* = 120, *U* = 64*P* and *m_l_* = –*m_r_* = 1. Under these settings, the electric field of the two transmitted beams are express as:

(16) $$E_{\mathrm{out}}^{I}\propto e^{i\left(-2\pi r/U\right)}\left[\begin{array}{c} \cos\left(-\theta -\gamma^{\mathrm{in}}\right)\\ \sin\left(-\theta -\gamma^{\mathrm{in}}\right) \end{array}\right]$$

(17) $$E_{\mathrm{out}}^{\mathrm{III}}\propto e^{i\left(-2\pi r/U\right)}\left[\begin{array}{c} \cos\left(-\theta -\gamma^{\mathrm{in}}+\pi /2\right)\\ \sin\left(-\theta -\gamma^{\mathrm{in}}+\pi /2\right) \end{array}\right]$$

**Figure 4:**
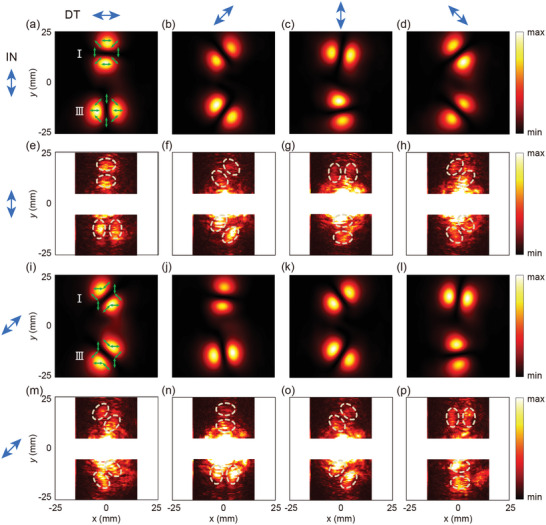
Theoretical prediction of the spatial polarization distribution and corresponding simulation and experimental results of M‐2B. (a–d) and (e–h) Simulated and experimental intensity components at polarization directions of 0, π/4, π/2, and 3π/4 under LP incidence with *γ* ^in^ = π/2, respectively. (i–l) and (m–p) Simulated and experimental intensity components at polarization directions of 0, π/4, π/2, and 3π/4 under LP incidence with *γ* ^in^ = π/4, respectively. The blue arrows in the left column represent the incident polarization (IN), while those in the row represent the detection polarization (DT). The green arrows indicate the designed polarization distributions of the output beams. The white dashed ellipse outlines the split‐lobe electric field pattern.

These equations illustrates that under LP incidence with orientation angles of *γ* ^in^ = π/2 and π/4, the polarization spatial distributions of the two transmitted beams are represented by green arrows in **Figure** **4**a,i, respectively. Electromagnetic field simulations for the M‐2B device were performed using the same parameters as those applied for the M‐4D device. For *γ* ^in^ = π/2, the simulated electric field intensity components at polarization directions of 0, π/4, π/2, and 3π/4 are shown in Figure 4a–d. Each beam exhibits clear symmetric split‐lobe patterns, characteristic of a cylindrical vector beam. For Beam‐I, at detection angles of 0 and π/2, the electric field lobes are strongest perpendicular to the detection polarization, with extinction occurring along the detection direction. This indicates that the localized polarization orientation is 0 at azimuthal angles *θ* = π/2 and 3π/2, and π/2 at azimuthal angles *θ* = 0 and π. In contrast, at detection angles of π/4 and 3π/4, the lobes align with the detection polarization, with extinction perpendicular to it. This indicates that the localized polarization is π/4 at azimuthal angles *θ* = π/4 and 5π/4, and 3π/4 at azimuthal angles *θ* = 3π/4 and 7π/4. The spatial polarization distribution aligns with the theoretical prediction of −*θ* − π/2, as derived from Equation (16). Furthermore, the electric field lobes of Beam‐III are consistently perpendicular to those of Beam‐I, confirming that the localized polarization of Beam‐III is expressed as −*θ*, as green arrows shown in Figure 4a. This consistency with the π/2 rotation between the two beams is demonstrated in Equations (16) and (17). For *γ* ^in^ = π/4, the simulated electric field intensity components at detection angles of 0, π/4, π/2, and 3π/4 were presented in Figure 4i–l. In this case, the spatial polarization distribution of Beam‐I changes to −*θ* − π/4, while that of Beam‐III changes to −*θ* + π/4. All detected electric field lobes of Beam‐I and Beam‐III are consistent with the spatial polarization distributions depicted as green arrows in Figure 4i.

M‐2B was fabricated, and its micrograph is presented in Figure 1h. Measurements were performed with the scanning area selected to avoid detecting the directly transmitted wave at the center, and the results are shown in Figure 4e–h,m–p. Overall, the experimental results are consistent with the simulations, further demonstrating the feasibility of the approach. However, it is worth noting that the strong intensity near the center is primarily due to the directly transmitted wave from the input beam, which distorts the output beam pattern. FP interference, fabrication inaccuracies and imperfect shape of the input beam are likely contributing factors to the less‐than‐ideal patterns (see Figures S15,S17 and S25, Supporting Information). Simulations and experiments were also conducted for *γ* ^in^ *=* 0 and *γ* ^in^ *=* 3π/4 (see Section S5, Supporting Information for details). The results show that the polarization spatial distribution of Beam‐I under *γ* ^in^ *=* 0 (or *γ* ^in^ *=* 3π/4) is identical to that of Beam‐III under *γ* ^in^ *=* π/2 (or *γ* ^in^ *=* π/4), and vice versa. In other words, rotating the incident polarization by π/2 swaps the polarization spatial distributions of the two transmitted beams. Specifically, as *γ* ^in^ changes to 0, π/4, π/2 and 3π/4, the spatial polarization distribution of Beam‐I alternates between matching the green arrows of Beam‐III in Figure 4a, Beam‐I in Figure 4i, Beam‐I in Figure 4a and Beam‐III in Figure 4i, respectively. Conversely, the polarization distribution of Beam‐III aligns with the green arrows of Beam‐I in Figure 4a, Beam‐III in Figure 4i, Beam‐III in Figure 4a and Beam‐I in Figure 4i, respectively. Additionally, under RCP (LCP) incidence, both Beam‐I and Beam‐III are LCP *m_l_*‐th (RCP *m_r_*‐th) Bessel beams. This approach is flexible and can be extended to more general cases. For instance, when *m_l_ =* −*m_r_ =* −1, the spatial polarization distributions of Beam‐I and Beam‐III are *θ* − *γ* ^in^ and *θ* − *γ* ^in^ + π/2, respectively. Specifically, for *γ* ^in^ *=* 0 (*γ* ^in^ *=* π/2), Beam‐I becomes a radially (azimuthally) polarized 0‐th Bessel beam, and Beam‐III an azimuthally (radially) polarized 0‐th Bessel beam (see Section S6, Supporting Information for details).

**Figure 5:**
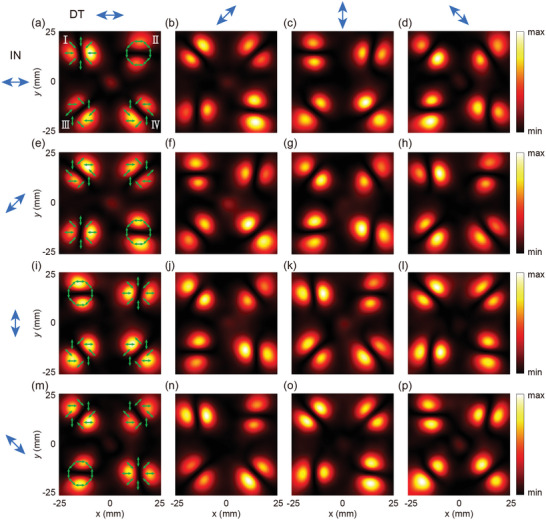
Theoretical prediction of the spatial polarization distribution and corresponding simulation results of M‐4B. (a–d), (e–h), (i–l), and (m–p) Electric field intensity components at polarization directions of 0, π/4, π/2, and 3π/4 under LP incidence with *γ* ^in^ = 0, π/4, π/2, and 3π/4, respectively, where the green arrows indicate the theoretically predicted spatial polarization distribution of each beam. The blue arrows in the left column represent the incident polarization (IN), while those in the top row represent the detection polarization (DT).

By combining the M‐4D phase distribution with the Bessel beam phase distribution, four distinct vector Bessel beams with unique spatial polarization distributions are generated (see Sections S7 and S8, Supporting Information for details). The resulting meta‐device, referred to as M‐4B, was specifically designed for this purpose. **Figure** **5** presents the theoretical prediction of the spatial polarization distribution alongside the simulated electric filed patterns. For *γ* ^in^ = 0, π/4, π/2, and 3π/4, Beam‐I exhibits polarization orientations of *θ* (radial), *θ* + 3π/4, *θ* + π/2 (azimuthal), and *θ* + π/4 Bessel beams, respectively, in alignment with the theoretical spatial polarization distribution. By adjusting the incident polarization orientation, the spatial polarization distributions of the four output vector Bessel beams can be flexibly controlled. For instance, it is possible to switch the output between radially polarized Bessel beams among the four beams. These characteristics have significant applications in systems requiring active control, offering enhanced capabilities for achieving a large depth of field, high lateral resolution in imaging, sensing, and particle manipulation. The proposed design strategy provides more versatile and flexible solutions for advanced TPSM meta‐devices and integrated active systems.

## 3 Conclusion

This work demonstrates the effectiveness of spin‐decoupled phase control engineering in enabling TPSM meta‐devices. By combining spin‐decoupled phase control with gradient grating phase design and using circular polarization multiplexing techniques, the meta‐device M‐4D based on all‐dielectric HR silicon pillars was successfully demonstrated. It converts an incident LP wave into four beams with distinct polarization orientations. Compared to previous methods that generate identical or symmetric polarization states, our approach significantly enhances the flexibility of polarization control, allowing for the generation of identical, distinct, symmetric, or asymmetric polarization orientations. Incorporating specialized phase design enables the generation of distinct vector beams. The meta‐device M‐2B, which produces two vector Bessel beams with flexibly controlled spatial polarization distributions by adjusting the incident LP orientation, was developed. Additionally, other meta‐devices were designed to generate four Bessel beams with distinct spatial polarization distributions. In summary, this work presents a method for modulating the polarization orientations and spatial distributions of multiple beams, with potential applications in multi‐polarization beam converters, multiplexing systems, and active control systems. The proposed approach facilitates the design of advanced TPSM meta‐devices, applicable to THz‐wave sensing, imaging, polarization analysis, particle manipulation, communication, and information processing. Although demonstrated in the THz‐wave regime, this method can be extended to other frequency ranges within the electromagnetic spectrum, thereby broadening its impact.

## 4 Experimental Section

**Figure 6:**
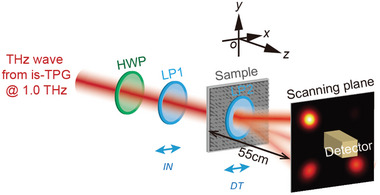
Experimental setup.

In the experimental setup, the THz source is an injection‐seeded THz‐wave parametric generator operating at 1.0 THz with *y*‐polarization.^[^ ^40^ ^]^ The THz beam was collimated into a quasi‐Gaussian profile (see Section S9, Supporting Information for details). It then sequentially passed through a THz half‐wave plate (HWP) operating at 1.0 THz and a linear polarizer (LP1) before passing through the center of the sample, as shown in **Figure** **6**. The HWP was used to rotate the polarization direction of the incident wave, while LP1 was used for additional polarization purification. Another linear polarizer (LP2) was placed directly behind the sample to detect different LP components of the THz wave generated by the sample, with a THz detector positioned approximately z = 55 mm behind the sample. The sample M‐4D was measured using a THz‐wave pyroelectric detector, which was scanned across the *x*‐*y* plane over a range of −25–25 mm in each direction, with a step size of 1 mm. To enhance detection sensitivity and spatial resolution during the measurement of M‐2B, a Schottky‐barrier‐diode (SBD) detector with a 1 mm aperture was used. Due to imperfections in the fabricated sample, the intensity of the directly transmitted central beam exceeded the SBD detector's damage threshold. To avoid direct detection of the central beam, the scanning parameters were set as follows: for Beam‐I, the scan spanned from −15 to 15 mm along the *x*‐axis and from 5 to 25 mm along the *y*‐axis; for Beam‐II, the scan ranged from −15 to 15 mm in the *x*‐direction and from −25 to −5 mm in the *y*‐direction. The step size for both scans was set to 0.5 mm.

## Conflict of Interest

The authors declare no conflict of interest.

## Supporting information



Supporting Information

## Data Availability

The data that support the findings of this study are available from the corresponding author upon reasonable request.
