# Supplementary material for: Terahertz‐Wave Polarization Space‐Division Multiplexing Meta‐Devices based on Spin‐Decoupled Phase Control
Source: Adv Sci (Weinh). 2024 Dec 30;12(8):2412688. doi: 10.1002/advs.202412688 (PMC11848590; doi:10.1002/advs.202412688)
Supplement: Supplementary file 1 — Supporting Information [file ADVS-12-2412688-s001.docx]

Supporting Information

Terahertz-Wave Polarization Space-Division Multiplexing Meta-Devices based on Spin-Decoupled Phase Control

*Yuehong Xu^*^, Yuma Takida, Tetsu Suzuki, and Hiroaki Minamide^*^*

**Section 1.** **Selection criteria for choosing 32 unit structures from the database**

The selection of 32 unit structures in the database was carried out through the following steps:

1. Initial Selection: Structures exhibiting half-wave plate properties were identified and filtered based on the following criterion: |Δ*ϕ*| < π ± 0.01π.
2. Transmission Amplitude Filtering: From the initial set, structures with higher transmission amplitudes in both polarization directions were further selected according to the criterion: *A_f_* > 0.66 and *A_s_* > 0.66.
3. Phase Gradient Optimization: Using a mean square error evaluation, structures whose *ϕ_f_* most closely matched the ideal 32-level phase gradient were chosen. This final selection formed the 32 unit structures used in this work.

This selection method ensures that the chosen structures retain half-wave plate properties while satisfying the requirement of covering a dynamic phase range of 0 to 2π.

**Section 2.** **Phase and transmission amplitude responses of the selected 32 basic units** **under CP incidence**

**Figure S1** shows the phase and transmission amplitude responses of the selected 32 basic units under CP incidence. Under both LCP and RCP incidence, the phase shifts of the cross-RCP and cross-LCP output waves of the selected units cover the entire phase range from 0 to 2π, as shown in Figure S1(a). Additionally, both the cross-RCP and cross-LCP output wave exhibit high transmission amplitude with average values of 0.8 compared to the co-LCP and co-RCP output wave, as shown in Figure S1 (b). The transmission amplitude of cross-RCP and cross-LCP output wave are equal. These phase and transmission responses indicate that the selected 32 structures function as quasi-half-wave plates. By combining the PB phase with these quasi-half-wave plates, the phases of the cross-RCP and cross-LCP output waves can be independently controlled.


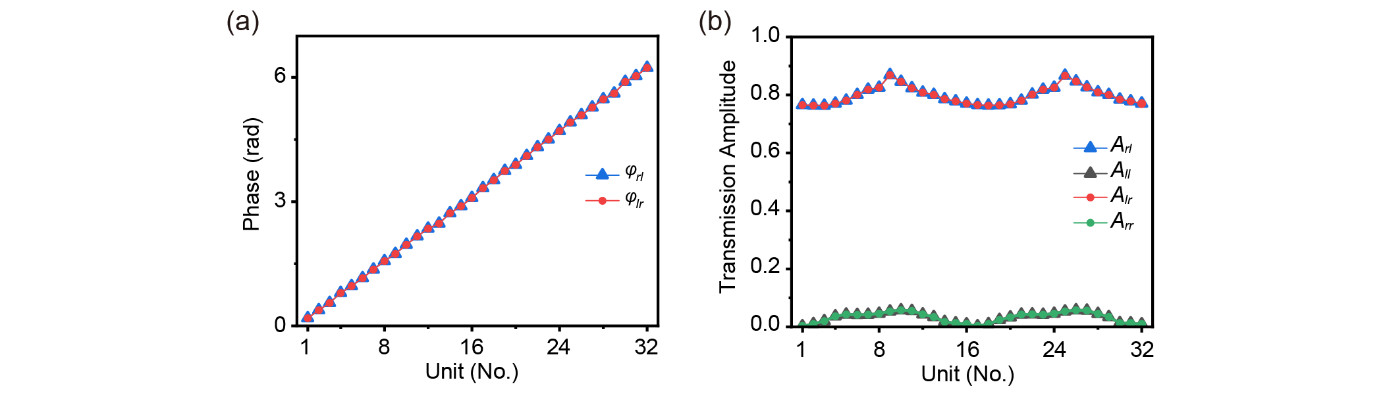


**Figure S1.** Phase and transmission amplitude responses of the selected 32 basic units with *α* = 0 under CP incidence at 1.0 THz. (a) Phase shift profiles of cross-RCP wave and cross-LCP wave. (b) Transmission amplitude profiles of cross- and co- RCP/LCP.

**Section 3. Parameters of silicon material used in HFSS simulations and actual device fabrication**

Both the substrate and the dielectric pillars are composed of the same material, with the substrate serving as structural support for the metasurface. The substrate’s phase contribution remains uniform across the metasurface and is polarization-independent, while the dielectric pillars introduce polarization-dependent phase modulation. In the HFSS simulation of the complete metasurface device, both the substrate and the dielectric pillars are modeled using silicon (Si) material from the HFSS library, with a relative permittivity of 11.9 and a dielectric loss tangent of 0. To optimize memory usage and reduce the computational domain, the substrate thickness was set to 100 µm, and the excitation port was placed on the underside of the substrate, ensuring that the substrate thickness had no effect on the simulation results.

For the actual device fabrication, a 1 mm-thick, undoped, intrinsic Si wafer was used, featuring a resistivity exceeding 20 kΩ·cm and a double-sided polished surface. The relative permittivity and dielectric loss tangent of the Si wafer were experimentally determined using a terahertz (THz) time-domain spectroscopy (THz-TDS) system. As shown in **Figure S2**, at 1.0 THz, the measured relative permittivity is 11.67, and the dielectric loss tangent is almost negligible (0.0002). These meta-devices were fabricated using deep reactive ion etching (DRIE) on this Si wafer.


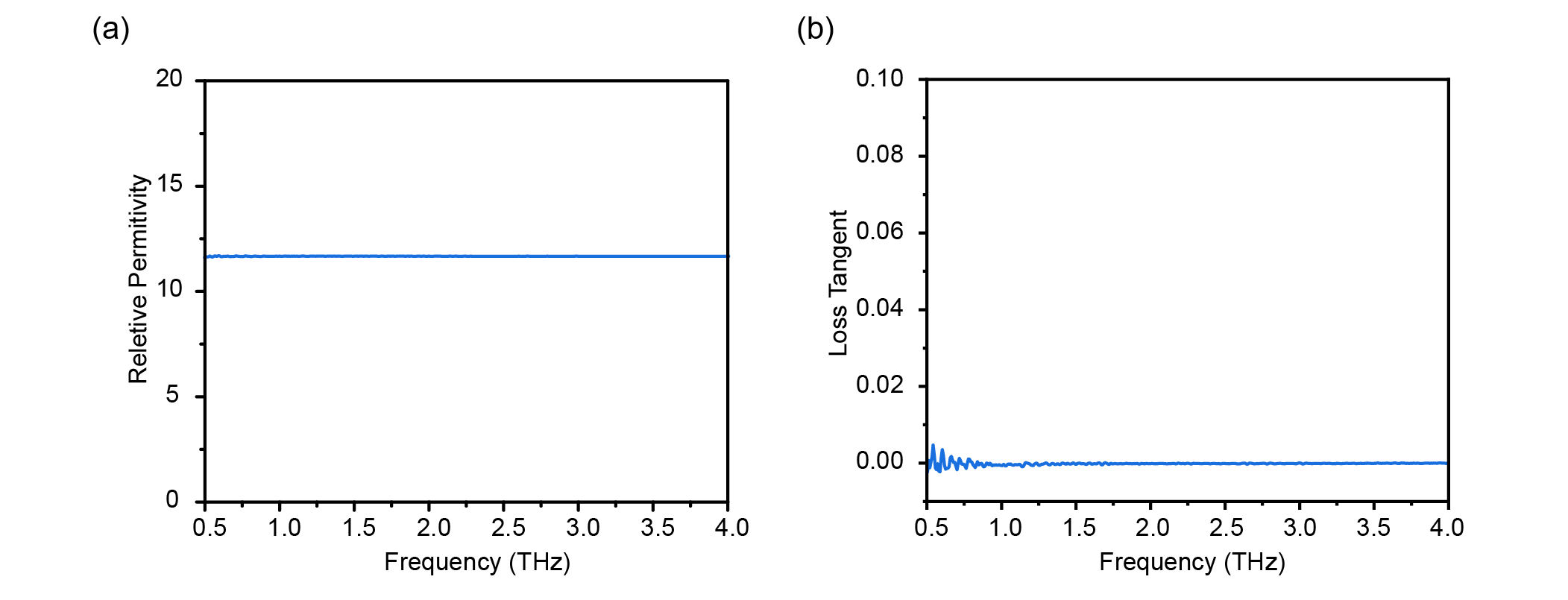


**Figure S2.** Parameters of the Si wafer measured using the THz TDS. (a) Relative permittivity. (b) Dielectric loss tangent.

**Section 4. Polarization ellipses at the center of output four beams under LP incidence with different polarization orientations**

**Figure S3** shows the simulated results of polarization ellipses at the center of Beams I, II, III, and IV under linearly polarized (LP) incidence with different polarization orientations. As shown in Figure S3(a), under LP incidence with orientation angle of *γ*^in^ = 0°, the four output beams are almost LP waves with polarization orientations of *γ*^I^ = 5.6°, *γ*^II^ = 100.9°, *γ*^Ⅲ^ = 39.9°, and *γ*^Ⅳ^ = 134.1°, respectively, and ellipticity of *χ*^I^ = 4.1°, *χ*^II^ = –5.7°, *χ*^III^ = 1.8°, and *χ*^IV^ = 8.1°, respectively. As shown in Figure S3(b), under LP incidence with orientation angle of *γ*^in^ = 45°, the four output beams are almost LP waves with polarization orientations of *γ*^I^ = 136.3°, *γ*^II^ = 48.8°, *γ*^Ⅲ^ = 179.1°, and *γ*^Ⅳ^ = 83.4°, respectively, and ellipticity of *χ*^I^ = 4.4°, *χ*^II^ = -7.9°, *χ*^III^ = –4.3°, and *χ*^IV^ = –5.4°, respectively. As shown in Figure S3(c), under LP incidence with orientation angle of *γ*^in^ = 90°, the four output beams are almost LP waves with polarization orientations of *γ*^I^ = 83.2°, *γ*^II^ = 174.6°, *γ*^Ⅲ^ = 141.3°, and *γ*^Ⅳ^ = 40.2°, respectively, and ellipticity of *χ*^I^ = –3.6°, *χ*^II^ = –4.4°, *χ*^III^ = –4.1°, and *χ*^IV^ = 5.4°, respectively. As shown in Figure S3(d), under LP incidence with orientation angle of *γ*^in^ = 135°, the four output beams are almost LP waves with polarization orientations of *γ*^I^ = 42.4°, *γ*^II^ = 137.3°, *γ*^Ⅲ^ = 93.9°, and *γ*^Ⅳ^ = 0.4°, respectively, and ellipticity of *χ*^I^ = –2.0°, *χ*^II^ = –3.5°, *χ*^III^ = 2.6°, and *χ*^IV^ = 8.0°, respectively. These results are essentially consistent with the theoretical design. It should be noted that there is slight discrepancy between the simulation results and the theoretical design. This may be attributed to the following reasons. One reason is the crosstalk issue between unit structures, caused by adjacent unit structures not being of the same structure. The unit structures obtained from the database assume a half-wave plate response on the condition that adjacent unit structures are identical, without considering the crosstalk effects in different adjacent unit structure configurations. Additionally, the selected unit structures are not perfect, and the unconverted co-CP components have a slight negative impact. This issue can be optimized by optimizing the structures. Another possible reason is that the HFSS simulation uses frequency-domain simulation, which may cause FP interference, negatively affecting the extracted polarization state. Furthermore, the polarization ellipse for each beam is reconstructed using the electric field data from a single pixel, which reduces the accuracy and signal-to-noise ratio of the polarization ellipses.


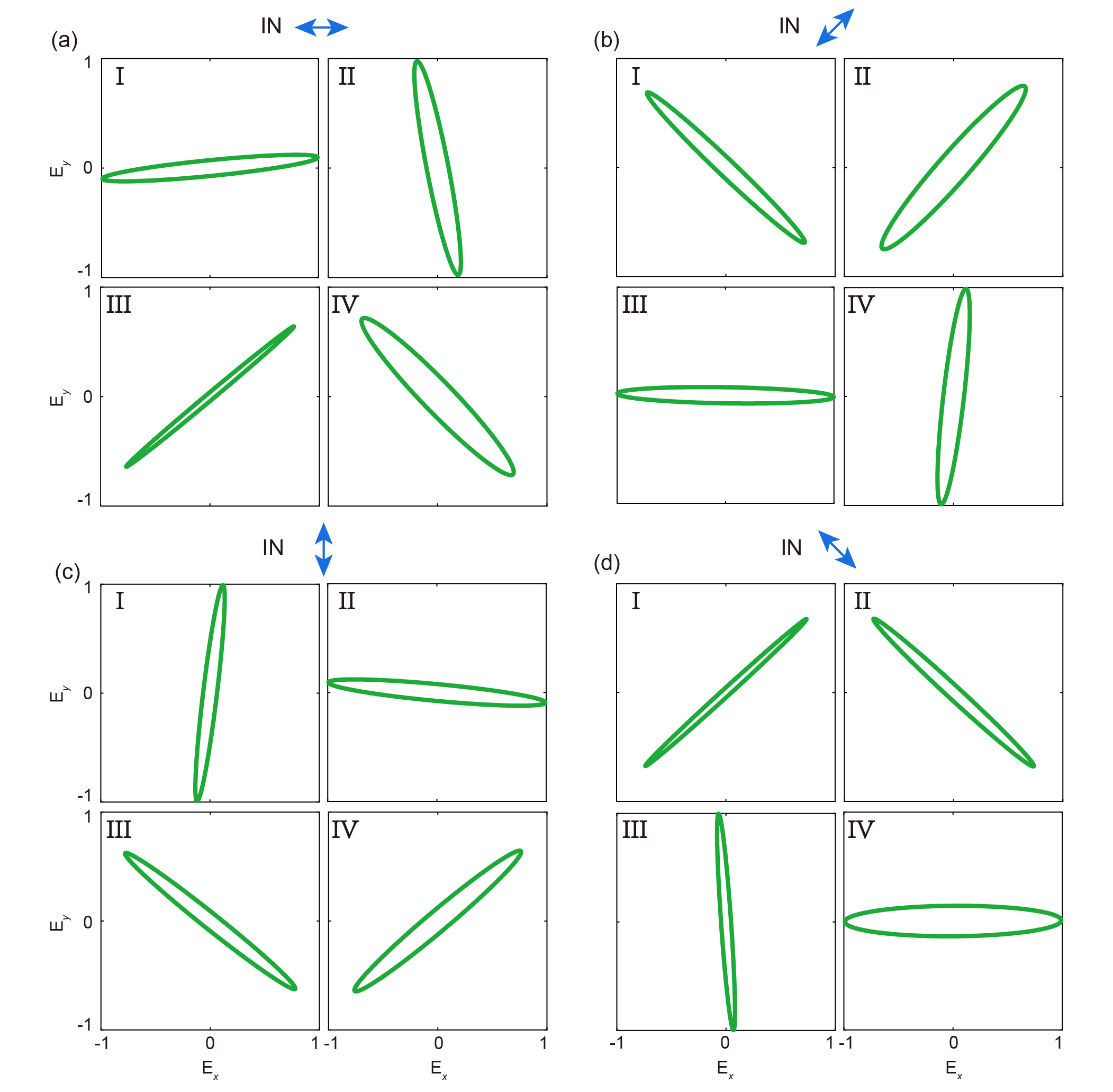


**Figure S3.** Simulated results of polarization ellipses at center of Beam I, II, III, and IV under LP incidence with *γ*^in^ = 0 (a), π/4 (b), π/2 (c), and 3π/4 (d). The top blue arrows represent the incident polarization direction (IN), while the Roman numerals represent the beam numbers.

**Section 5. Simulation and experimental results of M-2B for *γ*^in^ = 0 and 3π/4.**

Simulations and experiments of M-2B we re conducted for *γ*^in^ *=* 0 and *γ*^in^ *=* 3π/4. The results are shown in **Figure S4**. For *γ*^in^ *=* 0, the local polarization orientation of Beam-I is −*θ*, and Beam-III is −*θ* + π/2, as green arrows shown in Figure S4(a). For *γ*^in^ *=* 3π/4. the local polarization orientation of Beam-I is −*θ* + 3π/4, and Beam-III is −*θ* + π/4, as green arrows shown in Figure S4(i). The split-lobe patterns of electric field intensity components at 0, π/4, π/2, and 3π/4 polarization directions are aligning with the theoretical spatial polarization distribution. The polarization spatial distribution of Beam-I under *γ*^in^ *=* 0 (or *γ*^in^ *=* 3π/4) is identical to that of Beam-III under *γ*^in^ *=* π/2 (or *γ*^in^ *=* π/4), and vice versa.


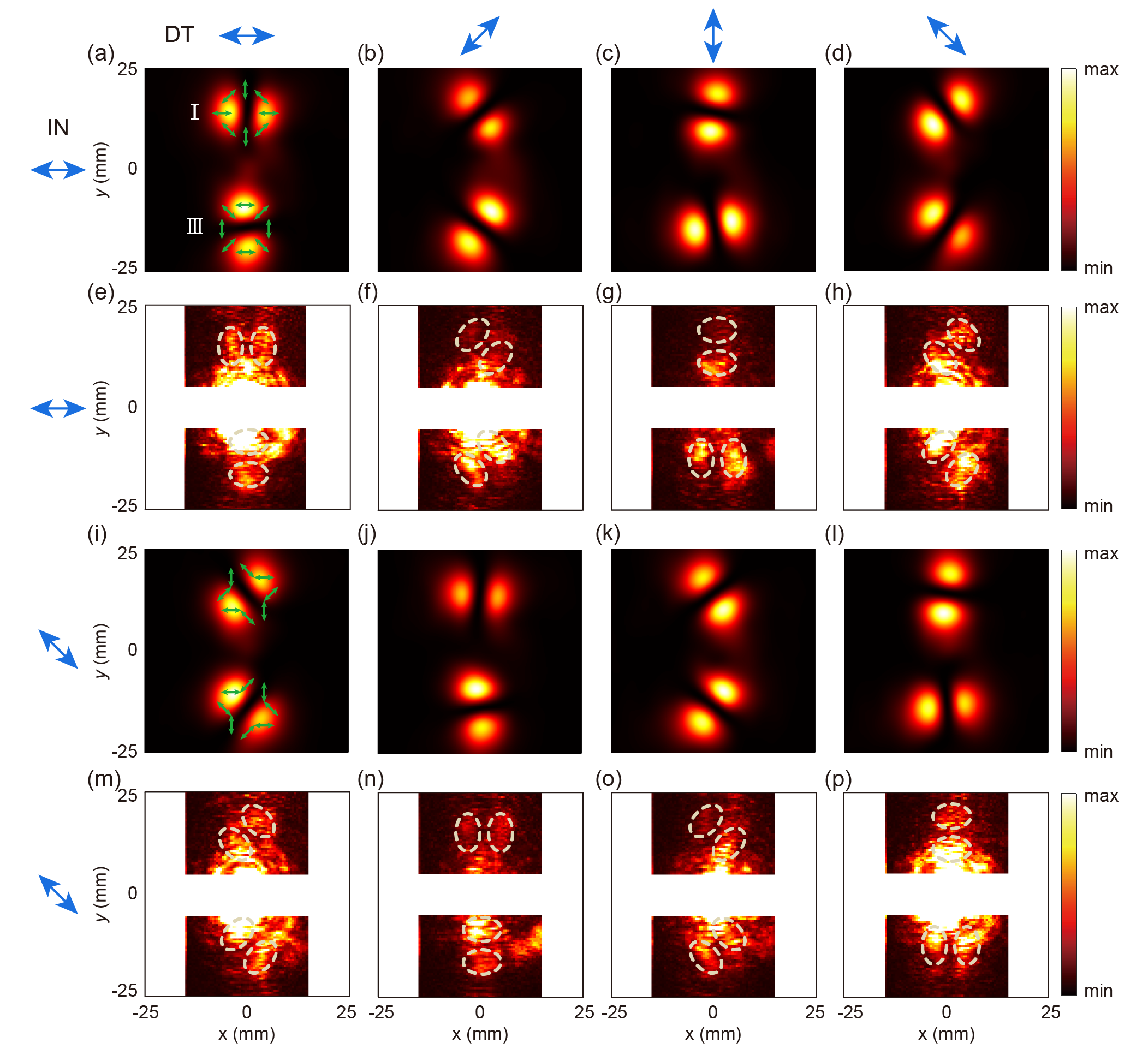


**Figure S4.** Theoretical prediction of the spatial polarization distribution and corresponding simulation and experimental results of M-2B. (a-d) and (e-h) Simulated and experimental intensity components at polarization directions of 0, π/4, π/2, and 3π/4 under LP incidence with *γ*^in^ = 0, respectively. (i-l) and (m-p) Simulated and experimental intensity components at polarization directions of 0, π/4, π/2, and 3π/4 under LP incidence with *γ*^in^ = 3π/4, respectively. The blue arrows in the left column represent the incident polarization (IN), while those in the row represent the detection polarization (DT). The green arrows indicate the designed polarization distributions of the output beams. The white dashed ellipse outlines the split-lobe electric field pattern.

**Section 6. Two-vector Bessel beam generators, denoted as M-2B-2**

Building on the design theory and parameters of M-2B, we designed the meta-device M-2B-2 by re-setting *m_l_ = –m_r_ = –*1. Consequently, Equation (15) is modified as

$E_{\text{out}}^{\Omega}\propto e^{i(-2\pi r/U)}\left[\begin{matrix} \cos(\theta+\gamma^{\Omega}) \\ \sin(\theta+\gamma^{\Omega}) \end{matrix}\right]$. (S1)

And the electric field of the two transmitted beams are express as

$E_{\text{out}}^{\text{I}}\propto e^{i(-2\pi r/U)}\left[\begin{matrix} \cos(\theta-\gamma^{\text{in}}) \\ \sin(\theta-\gamma^{\text{in}}) \end{matrix}\right]$, (S2)

$E_{\text{out}}^{\text{III}}\propto e^{i(-2\pi r/U)}\left[\begin{matrix} \cos(\theta-\gamma^{\text{in}}+\pi/2) \\ \sin(\theta-\gamma^{\text{in}}+\pi/2) \end{matrix}\right]$. (S3)

As a result, the green arrows in **Figure S5**(a), (e), (i), (m) shows the corresponding spatial polarization distribution of four beams for *γ*^in^ = 0, π/4, π/2, and 3π/4, respectively. The electric field intensity distribution at 55 mm from the metasurface is shown in Figure S5. Under LP incidence with orientation angle of *γ*^in^ = 0, the electric field intensity components at polarization directions of 0, π/4, π/2, and 3π/4 were extracted, as shown in Figure S5(a-d). For Beam-I, the electric field lobes are strongest along the detection polarization direction, indicating a radially polarized Bessel beam with a localized polarization orientation of *θ*. For Beam-III, the electric field lobes are strongest perpendicular to the detection polarization direction, indicating an azimuthally polarized Bessel beam with a localized polarization orientation of *θ* + π/2. Additionally, under LP incidence at orientation angles of *γ*^in^ = 0, π/4, π/2, and 3π/4, the simulation results show that Beam-I exhibits localized polarization orientations of *θ* (radially polarized), *θ* + 3π/4, *θ* + π/2 (azimuthally polarized), and *θ* + π/4 Bessel beams, respectively. All the simulation results are consistent with the theoretical predictions given by **Equation (S2)** and **S3**, as the green arrows shown.


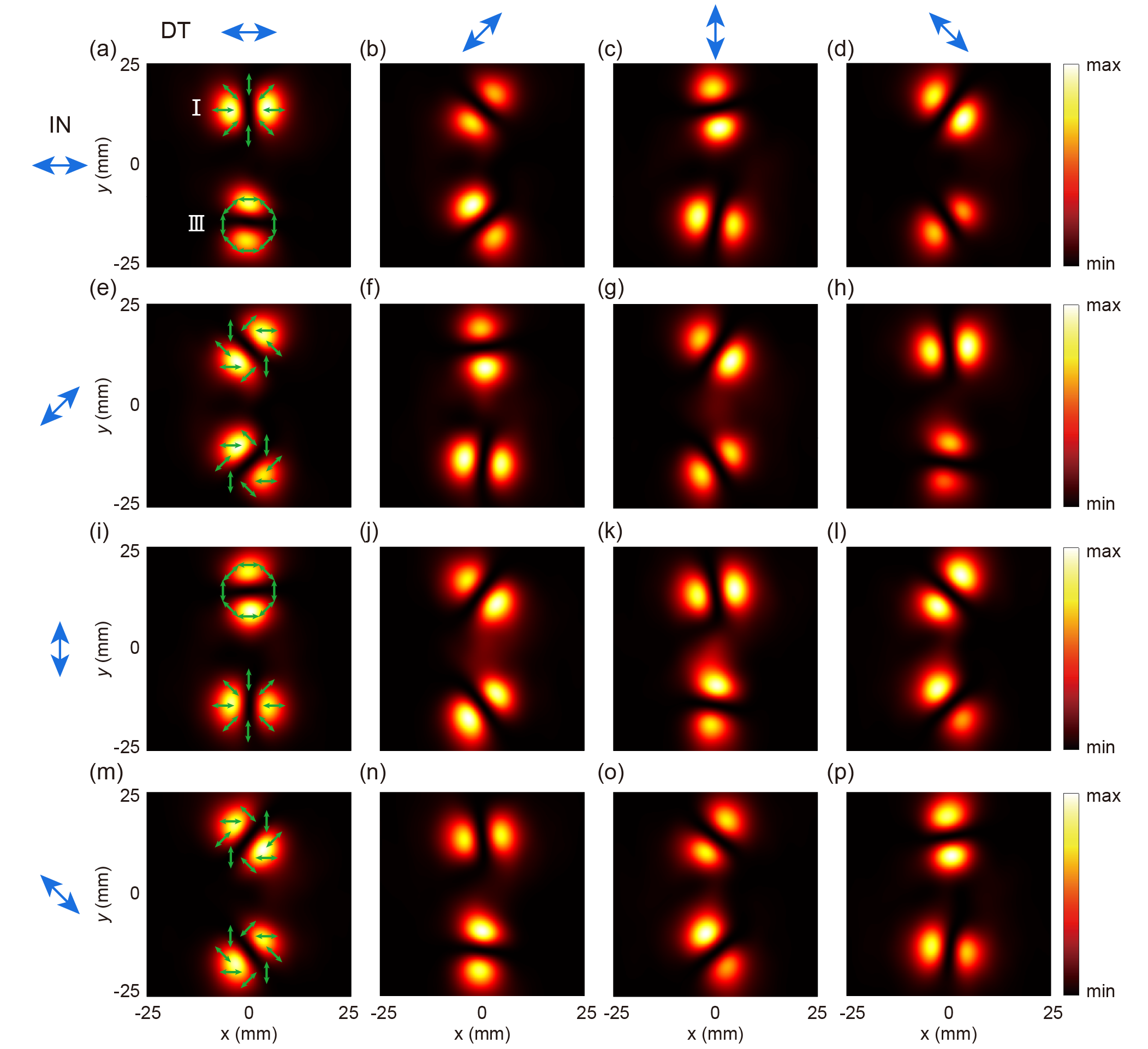


**Figure S5.** Theoretical prediction of the spatial polarization distribution and corresponding simulation results of M-2B-2. (a-d), (e-h), (i-l), and (m-p) Electric field intensity components at polarization directions of 0, π/4, π/2, and 3π/4 under LP incidence with *γ*^in^ = 0, π/4, π/2, and 3π/4, respectively, where the green arrows indicate the theoretically predicted spatial polarization distribution of each beam. The blue arrows in the left column represent the incident polarization (IN), while those in the top row represent the detection polarization (DT).

**Section 7. Four-vector Bessel beam generators,** **denoted as M-4B**

We propose the feasibility with meta-device M-4B, which generates four zero-order vector Bessel beams with different spatial polarization distributions. M-4B combines phase distribution of M-4D with an additional Bessel beam phase distribution. Under CP incidence, the modulated cross-RCP and cross-LCP phases by M-4B are designed as follows:

$\Psi_{lr}^{M-4B}=-2\pi r/U+m_{l}\theta+\Psi_{rl}^{M-4D}$, (S4)

$\Psi_{rl}^{M-4B}=-2\pi r/U+m_{r}\theta+\Psi_{lr}^{M-4D}$. (S5)

In this case, the four transmitted cross-RCP beams and the four output cross-LCP beams are all Bessel beams, with the LCP beams carrying OAM of *m_l_* and the RCP beams carrying OAM of *m_r_*. Under LP incidence with *γ*^in^, the electric field of transmitted Beam-Ω is express as

$E_{\text{out}}^{\Omega}\propto e^{i(-2\pi r/U)}\left[\begin{matrix} \cos(\frac{m_{r}-m_{l}}{2}\theta+\gamma^{\Omega}) \\ \sin(\frac{m_{r}-m_{l}}{2}\theta+\gamma^{\Omega}) \end{matrix}\right]$, (S6)

Where *γ^Ω^* represents the polarization rotation caused by the incident LP orientation angle and the gradient grating phase. Specifically, based on the gradient grating phase design of M-4D, the following equations apply: *γ*^I^ = −*γ*^in^, *γ*^II^ = −*γ*^in^ + π/2, *γ*^III^ = −*γ*^in^ + π/4, and *γ*^IV^ = −*γ*^in^ + 3π/4. The phase term of –2π*r*/*U* shows that the transmitted four beams are all Bessel beams carrying OAM of *m* = 0.

For meta-device M-4B, *m_l_ = –m_r_ =* –1 is set. Consequently, **Equation (S6)** is modified as

$E_{\text{out}}^{\Omega}\propto e^{i(-2\pi r/U)}\left[\begin{matrix} \cos(\theta+\gamma^{\Omega}) \\ \sin(\theta+\gamma^{\Omega}) \end{matrix}\right]$. (S7)

The Jones vector term shows that the localized polarization orientation is *θ* + *γ^Ω^*, indicating a rotation angle of *γ^Ω^* based on the azimuth angle of *θ*. And the electric field of the four transmitted beams are express as

$E_{\text{out}}^{\text{I}}\propto e^{i(-2\pi r/U)}\left[\begin{matrix} \cos(\theta-\gamma^{\text{in}}) \\ \sin(\theta-\gamma^{\text{in}}) \end{matrix}\right]$, (S8)

$E_{\text{out}}^{\text{II}}\propto e^{i(-2\pi r/U)}\left[\begin{matrix} \cos(\theta-\gamma^{\text{in}}+\pi/2) \\ \sin(\theta-\gamma^{\text{in}}+\pi/2) \end{matrix}\right]$, (S9)

$E_{\text{out}}^{\text{III}}\propto e^{i(-2\pi r/U)}\left[\begin{matrix} \cos(\theta-\gamma^{\text{in}}+\pi/4) \\ \sin(\theta-\gamma^{\text{in}}+\pi/4) \end{matrix}\right]$, (S10)

$E_{\text{out}}^{\text{IV}}\propto e^{i(-2\pi r/U)}\left[\begin{matrix} \cos(\theta-\gamma^{\text{in}}+3\pi/4) \\ \sin(\theta-\gamma^{\text{in}}+3\pi/4) \end{matrix}\right]$. (S11)

As a result, the green arrows in Figure 5(a), (e), (i), (m) shows the corresponding spatial polarization distribution of four beams for *γ*^in^ = 0, π/4, π/2, and 3π/4. Here, we performed electromagnetic field simulations of M-4B under the condition of *U* = 64*P*, with the same simulation settings as M-4D. The electric field intensity distribution at 55 mm from the metasurface is shown in Figure 5.

Under LP incidence with orientation angle of *γ*^in^ = 0, the electric field intensity components at polarization directions of 0, π/4, π/2, and 3π/4 were extracted, as shown in Figure 5(a-d). Clear split-lobe patterns are observed in each beam, indicating vector beams carrying OAM with *m* = 0.^[1]^ For Beam-I, the electric field lobes are strongest along the detection polarization direction, indicating a radially polarized Bessel beam with a localized polarization orientation of *θ*. For Beam-II, the electric field lobes are strongest perpendicular to the detection polarization direction, indicating an azimuthally polarized Bessel beam with a localized polarization orientation of *θ* + π/2. For Beam-III and Beam-IV, the electric field lobes are strongest along the direction rotated by π/4 and 3π/4 from the detection polarization direction, indicating a Bessel beam with a localized polarization orientation of *θ* + 3π/4 and *θ* + π/4. These results are consistent with the theoretical predictions given by Equation (11). Additionally, under LP incidence at orientation angles of *γ*^in^ = 0, π/4, π/2, and 3π/4, the simulation results show that Beam-I exhibits localized polarization orientations of *θ* (radially polarized), *θ* + 3π/4, *θ* + π/2 (azimuthally polarized), and *θ* + π/4 Bessel beams, respectively. Similarly, the localized polarization orientation of the other three beams undergoes a rotation of –*γ*^in^ with changes in the orientation angle of the incident LP wave, as *γ^Ω^* includes the factor of –*γ*^in^. Therefore, by adjusting the incident polarization orientation, the localized polarization orientations of the four output vector Bessel beams can be flexibly controlled. For instance, it is possible to switch the output between radially polarized Bessel beams among the four beams. These characteristics have significant applications in systems requiring active control and provide enhanced capabilities for achieving a large depth of field and high lateral resolution in imaging and sensing. The proposed design strategy provides more versatile and flexible solutions for advanced THz-wave polarization space-division multiplexing meta-devices and integrated active systems.

**Section 8.** **Four-vector Bessel beam generators, denoted as M-4B-2**

Building on the design theory and parameters of M-4B, we designed the meta-device M-4B-2 by re-setting *m_l_ = –m_r_ =* 1. Consequently, Equation (S6) is modified as

$E_{\text{out}}^{\Omega}\propto e^{i(-2\pi r/U)}\left[\begin{matrix} \cos(-\theta+\gamma^{\Omega}) \\ \sin(-\theta+\gamma^{\Omega}) \end{matrix}\right]$. (S12)

And the electric field of the four transmitted beams are express as

$E_{\text{out}}^{\text{I}}\propto e^{i(-2\pi r/U)}\left[\begin{matrix} \cos(-\theta-\gamma^{\text{in}}) \\ \sin(-\theta-\gamma^{\text{in}}) \end{matrix}\right]$, (S13)

$E_{\text{out}}^{\text{II}}\propto e^{i(-2\pi r/U)}\left[\begin{matrix} \cos(-\theta-\gamma^{\text{in}}+\pi/2) \\ \sin(-\theta-\gamma^{\text{in}}+\pi/2) \end{matrix}\right]$, (S14)

$E_{\text{out}}^{\text{III}}\propto e^{i(-2\pi r/U)}\left[\begin{matrix} \cos(-\theta-\gamma^{\text{in}}+\pi/4) \\ \sin(-\theta-\gamma^{\text{in}}+\pi/4) \end{matrix}\right]$, (S15)

$E_{\text{out}}^{\text{IV}}\propto e^{i(-2\pi r/U)}\left[\begin{matrix} \cos(-\theta-\gamma^{\text{in}}+3\pi/4) \\ \sin(-\theta-\gamma^{\text{in}}+3\pi/4) \end{matrix}\right]$. (S16)

As a result, the green arrows in **Figure S6**(a), (e), (i), (m) shows the corresponding spatial polarization distribution of four beams for *γ*^in^ = 0, π/4, π/2, and 3π/4. The electric field intensity distribution at 55 mm from the metasurface is shown in Figure S6. The split-lobe patterns of electric field intensity components at 0, π/4, π/2, and 3π/4 polarization directions are aligning with the theoretical spatial polarization distribution.


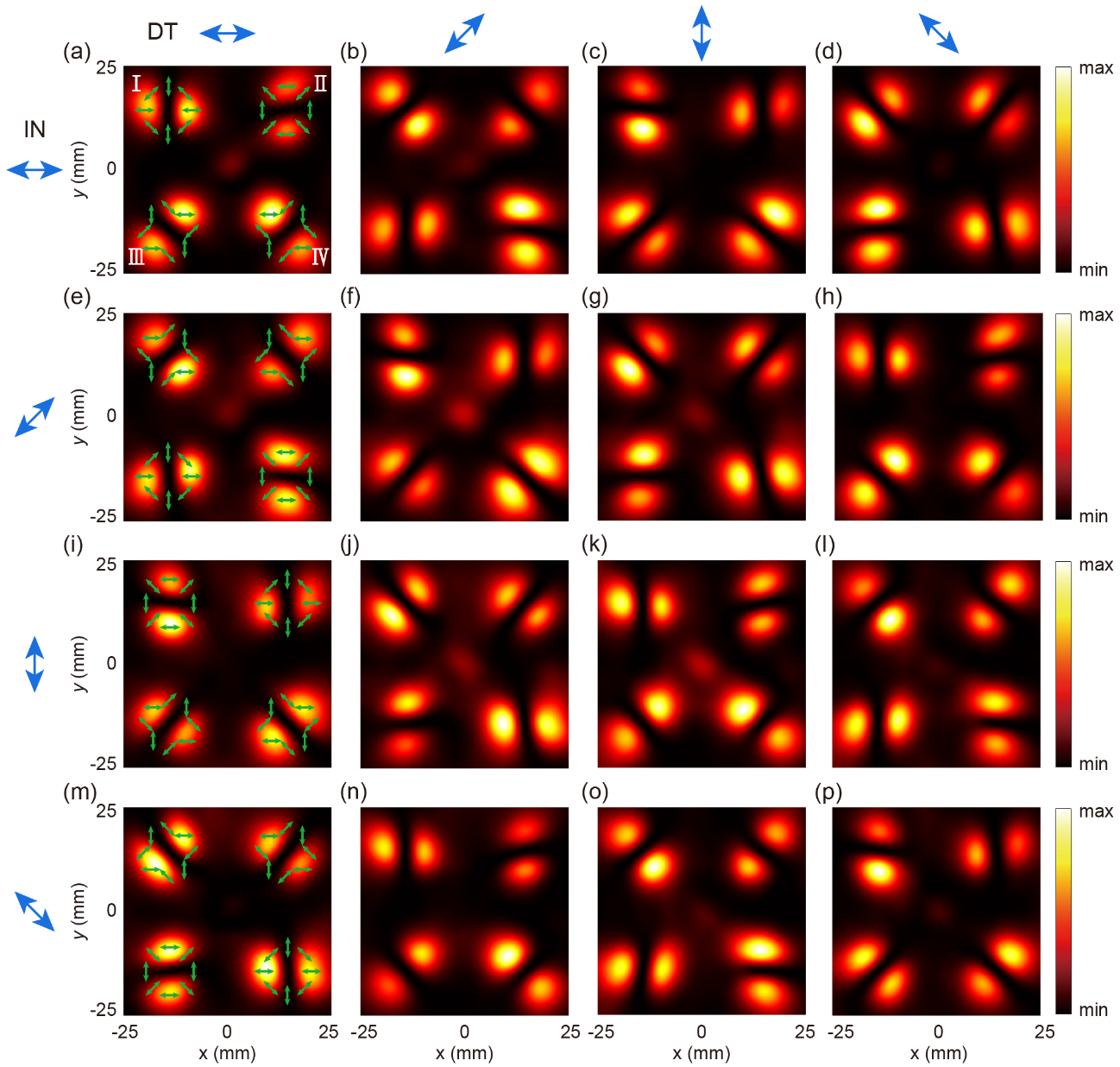


**Figure S6.** Theoretical prediction of the spatial polarization distribution and corresponding simulation results of M-4B-2. (a-d), (e-h), (i-l), and (m-p) Electric field intensity components at polarization directions of 0, π/4, π/2, and 3π/4 under LP incidence with *γ*^in^ = 0, π/4, π/2, and 3π/4, respectively, where the green arrows indicate the theoretically predicted spatial polarization distribution of each beam. The blue arrows in the left column represent the incident polarization (IN), while those in the top row represent the detection polarization (DT).

**Section 9.** **Experimental setup and THz beam profile**

**Figure S7** illustrates the experimental setup, which employs an injection-seeded THz-wave parametric generator (is-TPG) as the THz source.^[2]^ The THz waves are generated through efficient parametric wavelength conversion of sub-nanosecond pump beam in a MgO:LiNbO_3_ crystal. By simultaneously adjusting the incidence angle and wavelength of the seed beam according to the noncollinear phase-matching condition, the wavelength/frequency of the generated monochromatic THz waves can be tuned. In this system, the pump laser (depicted in dark green) operates with the following parameters: a wavelength of 1064 nm, a pulse width of 509 ps, a repetition rate of 30 Hz, and a pulse energy of 10 mJ. The seed laser (depicted in light green) is a continuous-wave laser with a tunable wavelength range of 1067 –1069 nm and an average output power of approximately 500 mW. The generated THz waves are extracted using a Si prism coupler after frequency conversion in the crystal, and are shaped into a collimated quasi-Gaussian beam using a cylindrical lens. The THz beam is then focused and collimated by two lenses and passes through THz polarization control components (a half-wave plate (HWP) and a linear polarizer (LP1)) before being directed onto the sample. The THz waves modulated by the sample pass through another linear polarizer (LP2) and reach the detection plane, where *x-y* plane scanning is performed using a THz detector. In this experiment, the central frequency of the generated THz wave was set to 1.0 THz, with a *y*-polarized orientation (*γ*^in^ = π/2).

In the absence of both the sample and LP2, the THz beam was measured using a THz pyroelectric detector. The *x-z* horizontal cross-section (*y* = 0) was scanned pixel by pixel with a step size of 1 mm, covering *x* from -15 mm to 15 mm and *z* from 0 mm to 50 mm. Similarly, the *y-z* vertical plane (*x* = 0 mm) was scanned, covering *y* from −15 mm to 15 mm and *z* from 0 mm to 50 mm. Additionally, *x-y* cross-sectional scans were performed at *z* = 0 mm and *z* = 50 mm, covering *x* and *y* ranges of −15 mm to 15 mm with 1-mm step sizes. The results are shown in **Figure S8**. At *z* = 0 mm, the full width at half maximum (FWHM) of the THz beam spot was approximately 6 mm in the *x*-direction and 5 mm in the *y*-direction. At *z* = 50 mm, the FWHM increased to approximately 8 mm in the *x*-direction and 7 mm in the *y*-direction, indicating slight divergence along the propagation direction.

During sample measurements, it was necessary to employ various incident and detected polarization states. For this purpose, a HWP and a LP1 were placed in the THz optical path before the sample to adjust the polarization directiont. The 0° position of the HWP corresponds to its fast axis aligned along the *y*-direction, while the 0° position of LP1 corresponds to the transmission axis aligned with the *x*-polarization. By rotating the HWP and LP1 to specific angle combinations—(45°, 0°), (22.5°,45°), (0°,90°) and (67.5°,135°)—incident linearly polarized waves with polarization angles of *γ*^in^ = 0, π/4, π/2, and 3π/4, respectively, were generated. On the detection side, LP2 was placed closely after the sample to analyze the polarization components of the THz pattern. The 0° position of LP2 corresponds to its transmission axis aligned with the *x*-polarization. Combined with the THz detector located after LP2, the LP2 angle was rotated to 0°, 45°, 90° and 135° to measure the polarization components corresponding to *γ*^out^ = 0, π/4, π/2, and 3π/4, respectively.


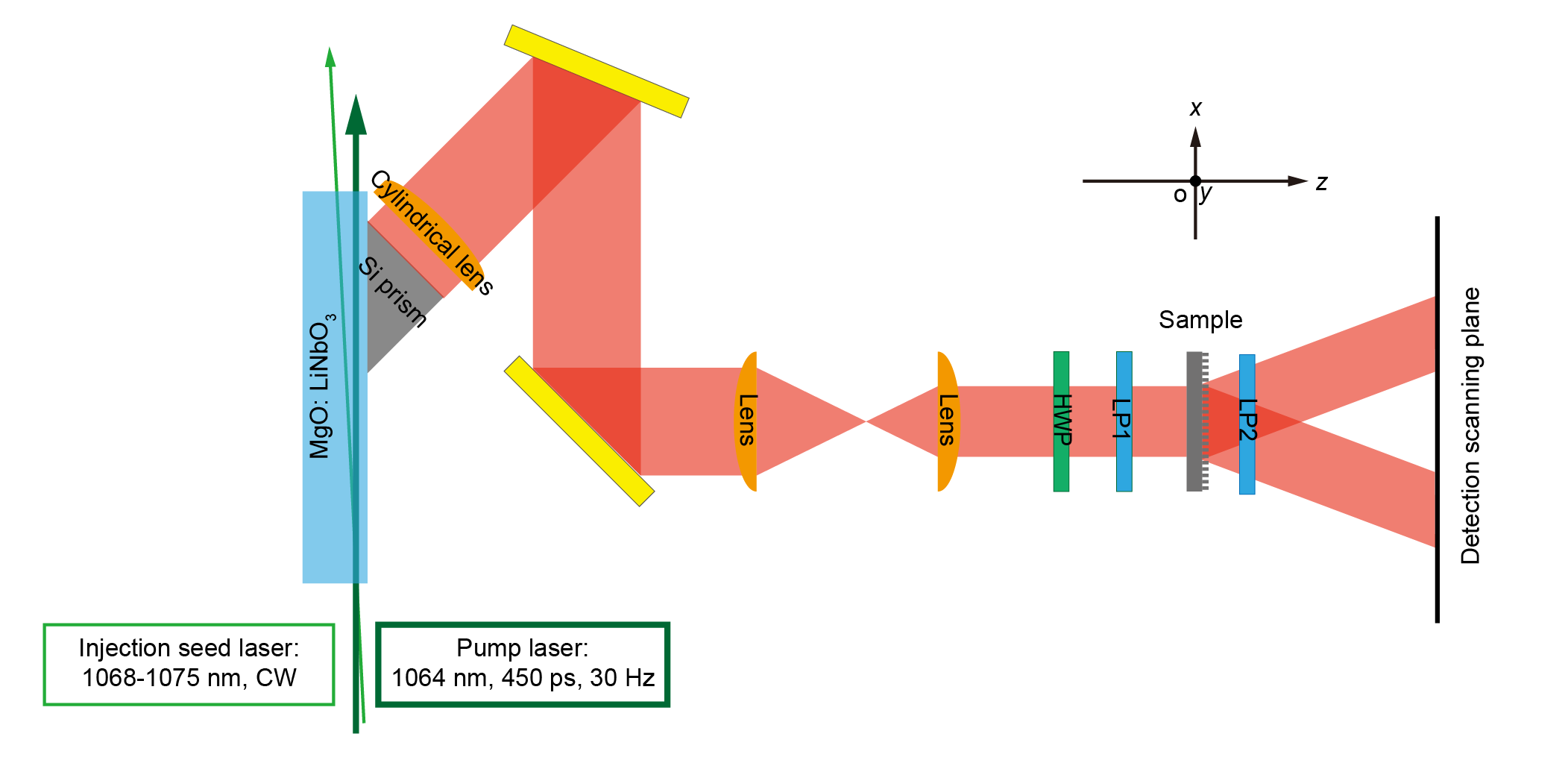


**Figure S7.** Schematic diagram of the experimental setup. The dark green, light green, and red paths represent the pump laser, injection seed laser, and generated terahertz wave, respectively.


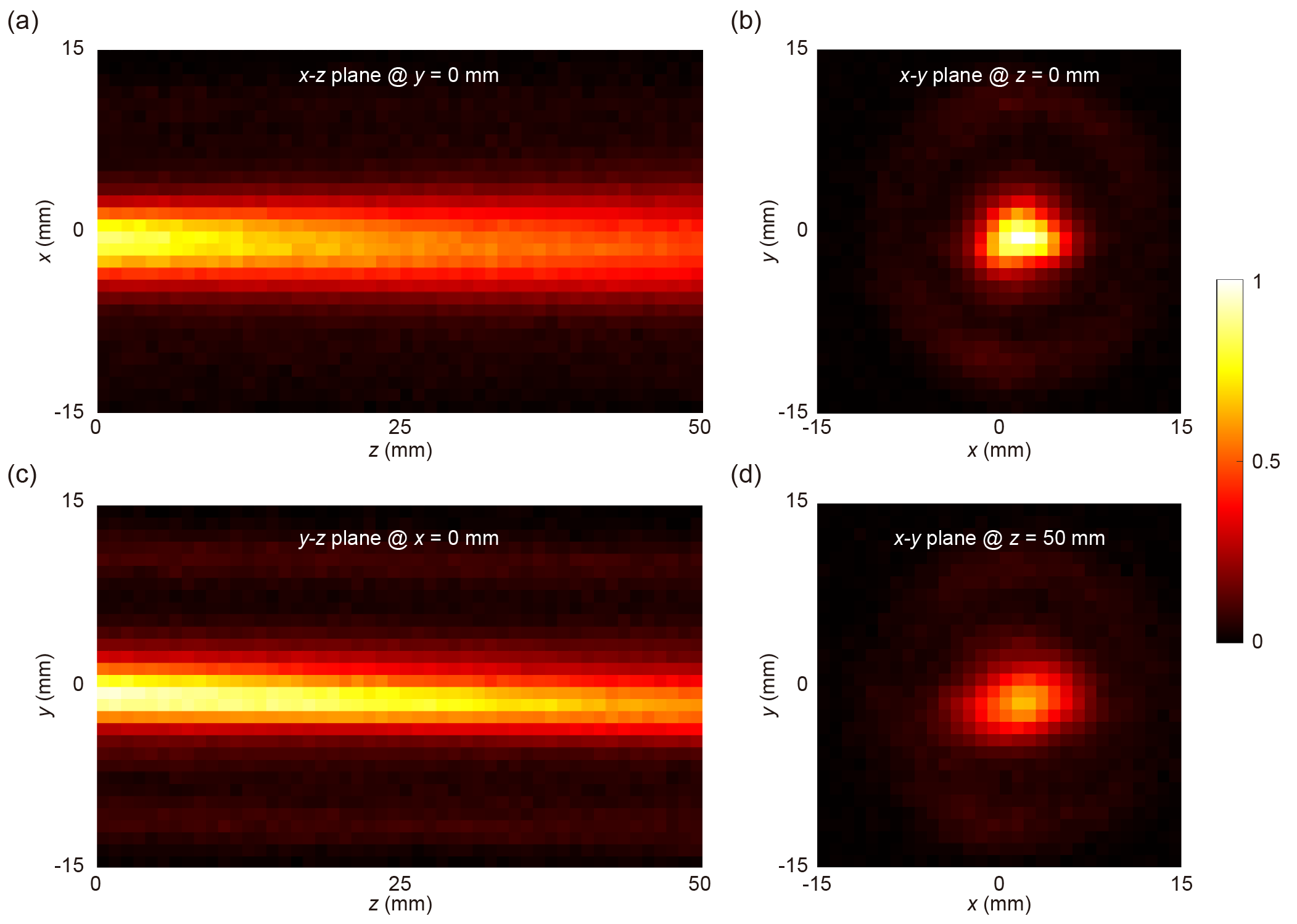


**Figure S8.** THz beam profile in the experimental setup. (a), (c) Measured propagation of the THz beam profile in the *x-z* horizontal plane (*y* = 0 mm) and *y-z* horizontal plane (*x* = 0 mm), respectively. (b), (d) Measured propagation cross-section of the THz beam in the *x-y* plane (z = 0 mm) and *x-y* plane (z = 50 mm), respectively.

**Section 10. Analysis of the impact of** **Fabry-Pérot interference**

The total thickness of the fabricated samples, including M-4D and M-2B, is 1 mm. One side of the sample is the metasurface interface, while the other is the polished Si interface. Reflections occur at both the metasurface and Si interfaces, resulting in multiple reflections between the two surfaces and consequently inducing Fabry-Pérot (FP) interference. To simplify the analysis of FP interference, the FP effect in a bare Si slab without a metasurface interface was investigated. The transmission amplitude at 1.0 THz was electromagnetically simulated for Si slabs with varying thickness *d*, as represented by the dash-dot purple line in **Figure S9**. The numerical calculation results, depicted by the blue solid line, demonstrate excellent agreement with the simulation. Due to the FP interference, the substrate thickness modulates the transmission amplitude, and the resonance frequency spacing caused by FP interference is expressed as Δ*f* = c/(2*nd*) , where c = 3×10^8^m/s is the speed of light in vacuum, and *n* is the refractive index of the slab. Smaller Δ*f* implies rapid oscillations in the frequency domain, resulting in larger variations in transmission amplitude, making it highly sensitive to frequency. Thinner substrates lead to larger Δ*f*, as represented by the brown solid line in Figure S9, reducing the sensitivity of transmission to frequency. Thus, varying the substrate thickness leads to differing degrees of impact on device performance, driven by the combined effects of FP interference from the substrate and complex reflection/transmission modulation at the metasurface interface.

A wideband measurement of the central beam directly transmitted through the M-4D sample was performed using a frequency sweep system,^[2]^ and the results are shown as the blue curve in **Figure S10**(a). FP oscillations are observed. A numerical fitting of the FP oscillations for a Si slab with a thickness of 0.82 mm is shown as the red curve. The six peak frequencies extracted from both the sample and the Si slab are compared in Figure S10(b), showing excellent agreement. To further investigate the impact of FP interference on the overall device performance, electromagnetic simulations were conducted with FP interference considered. In these simulations, an air gap is introduced between the excitation source port and the bottom surface of the substrate, unlike the simulations in the main text, where the excitation source port was placed directly on the substrate's bottom surface. Due to memory limitations in electromagnetic simulations, it was not feasible to simulate the full 1 mm-thick sample or the 0.82 mm-thick substrate. Instead, a 50 µm-thick substrate was chosen as an example for simulation, given that Si slabs with thicknesses of 50 µm and 0.82 mm exhibit comparable transmission amplitudes, as indicated by the blue stars in Figure S9. This setup allowed for an analogous observation. The simulation results are shown in **Figure S11**, where FP interference significantly reduces the overall device performance due to multiple complex reflections/transmissions between the metasurface and the substrate surface. This behavior is consistent with the experimentally observed degradation in performance (Figure 3). And simulations of meta-device M-2B were conducted considering FP interference with 50 µm thicknesses substrates, as shown in **Figure S15**. To examine the influence of different substrate thicknesses on the final performance, simulations were also conducted for substrates with thicknesses of 43 µm, 65 µm, and 100 µm, as indicated by the black stars in Figure S9. The corresponding results are shown in **Figure S12**, **S13** and **S14**. The results demonstrate that substrate thickness significantly affects the device’s performance.

The degradation in device performance caused by FP interference can be addressed by introducing an anti-reflection structure on the bottom surface of the substrate, eliminating the reflection and thus suppressing FP interference. As shown by the red solid line in Figure S9, while one side of the Si slab has zero reflectivity, the calculated transmission amplitude remains constant at 0.83, independent of the slab thickness. Alternatively, using a substrate material with a lower refractive index, such as quartz with *n* ≈ 1.5, can partially mitigate FP interference, as indicated by the green solid line in Figure S9. Furthermore, while the device is used in a time-domain spectroscopy system, FP interference can be effectively suppressed by truncating the delayed reflected signal in the time-domain data.

In summary, FP interference-induced performance degradation can be eliminated by introducing anti-reflection layers on the bottom substrate surface or be mitigated by using substrate materials with a lower refractive index. In time-domain systems, the delayed multi-reflected signal from the thick substrate can be truncated to eliminate the influence of FP interference.


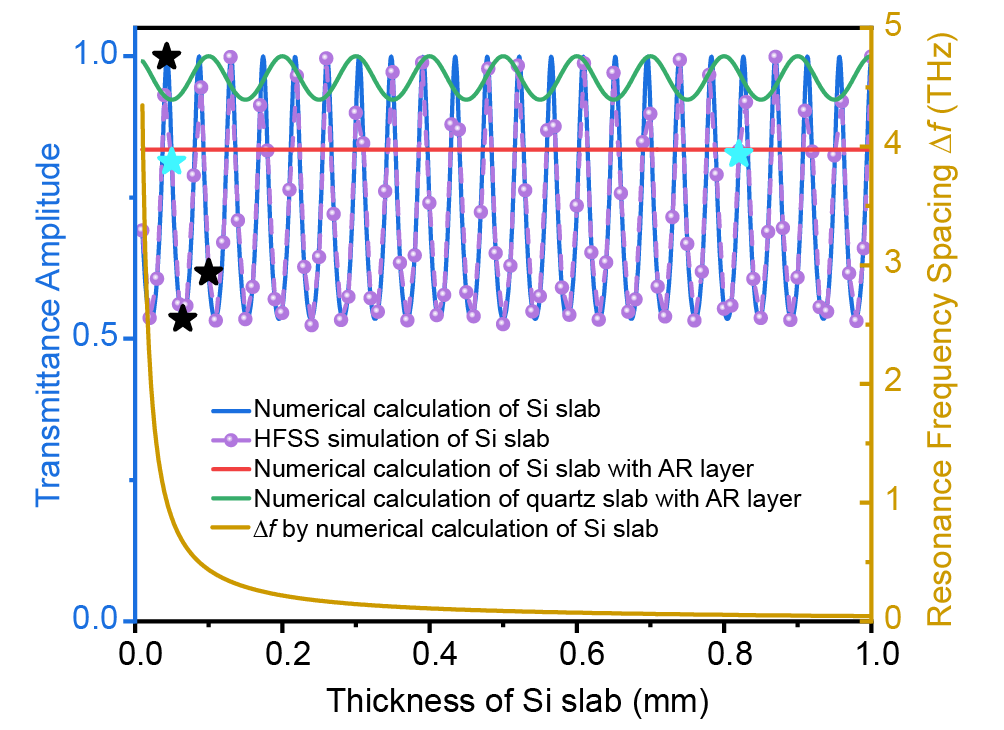


**Figure S9.** FP interference effect within a bare Si slab without a metasurface interface at 1.0 THz.


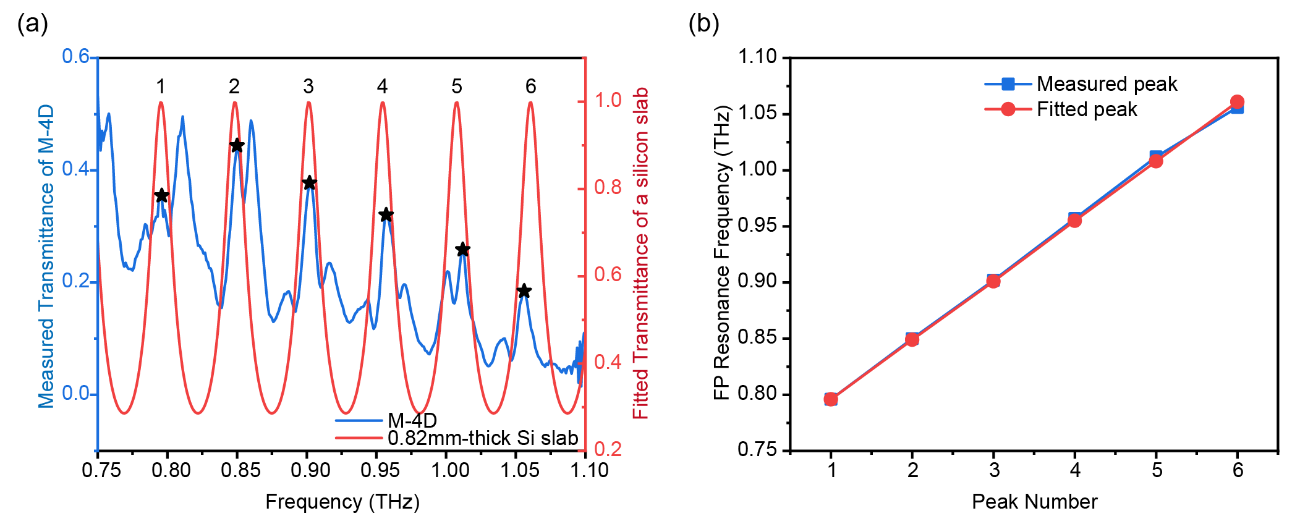


**Figure S10.** Broadband transmittance analysis and FP resonance peak analysis of the central beam transmitted directly through the M-4D sample. (a) Comparison of the measured transmittance with the Si slab fitting. (b) Comparison of the measured FP resonance frequencies with the Si slab fitting.


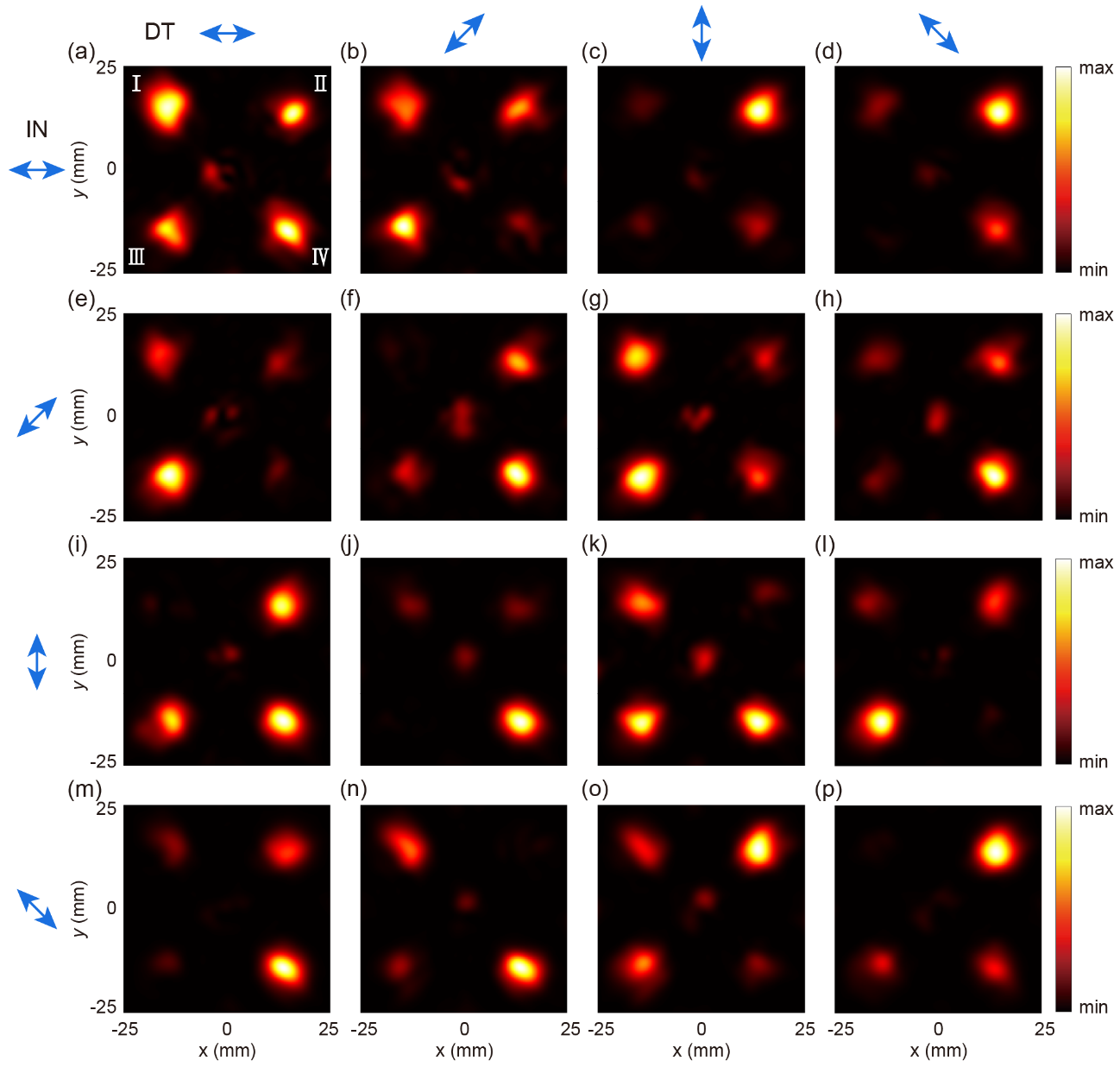


**Figure S11.** Electromagnetic simulation results of the M-4D sample, considering FP interference with a substrate thickness of 50 μm. (a-d), (e-h), (i-l), and (m-p) Electric field intensity components at 0, π/4, π/2, and 3π/4 polarization directions under LP incidence with *γ*^in^ = 0, π/4, π/2, and 3π/4, respectively, where the green arrows indicate theoretically predicted polarization direction of each beam. The blue arrows in the left column represent the incident polarization (IN), while those in the top row represent the detection polarization (DT).


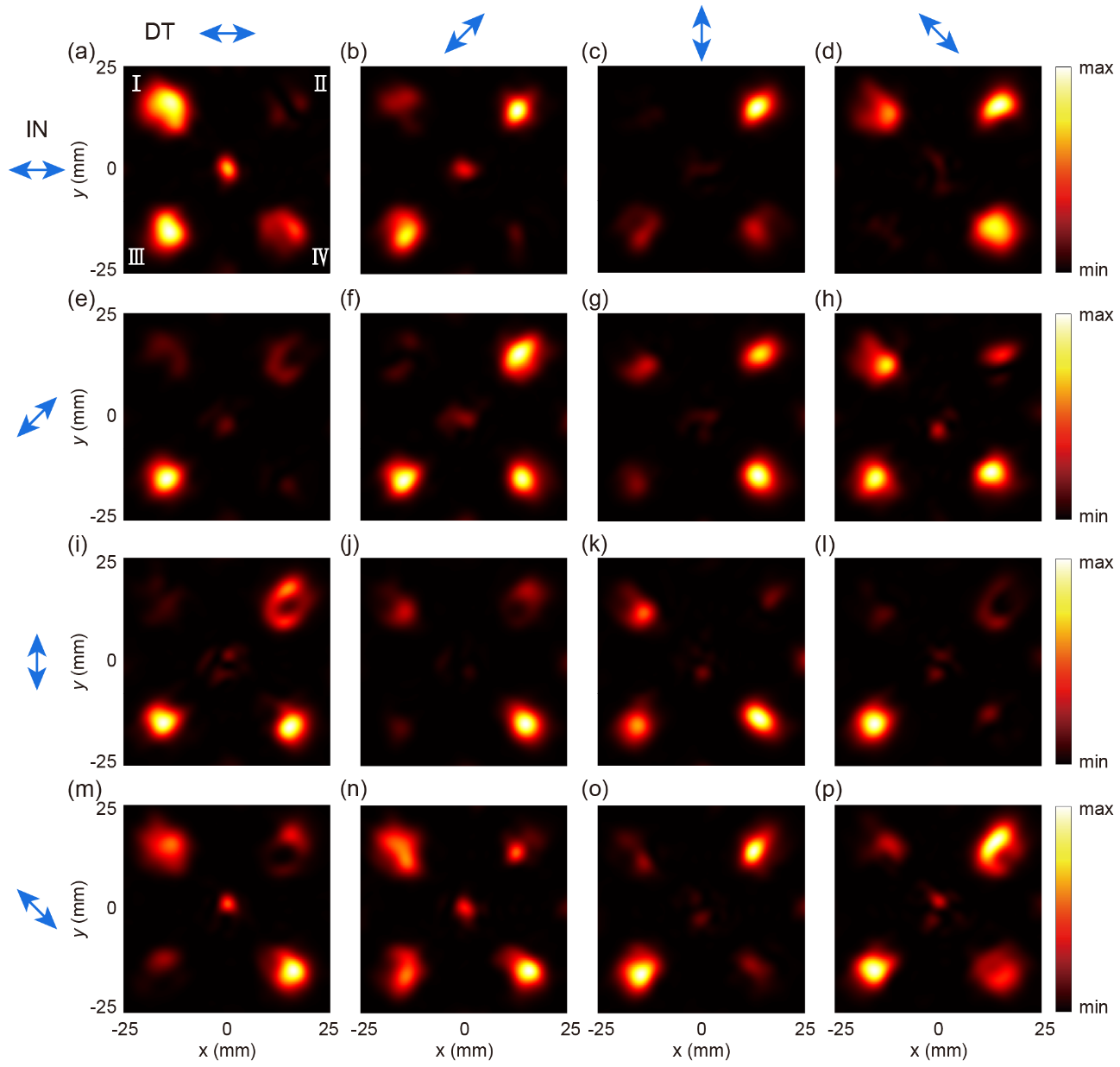


**Figure S12.** Electromagnetic simulation results of the M-4D sample, considering FP interference with a substrate thickness of 43 μm. (a-d), (e-h), (i-l), and (m-p) Electric field intensity components at 0, π/4, π/2, and 3π/4 polarization directions under LP incidence with *γ*^in^ = 0, π/4, π/2, and 3π/4, respectively, where the green arrows indicate theoretically predicted polarization direction of each beam. The blue arrows in the left column represent the incident polarization (IN), while those in the top row represent the detection polarization (DT).


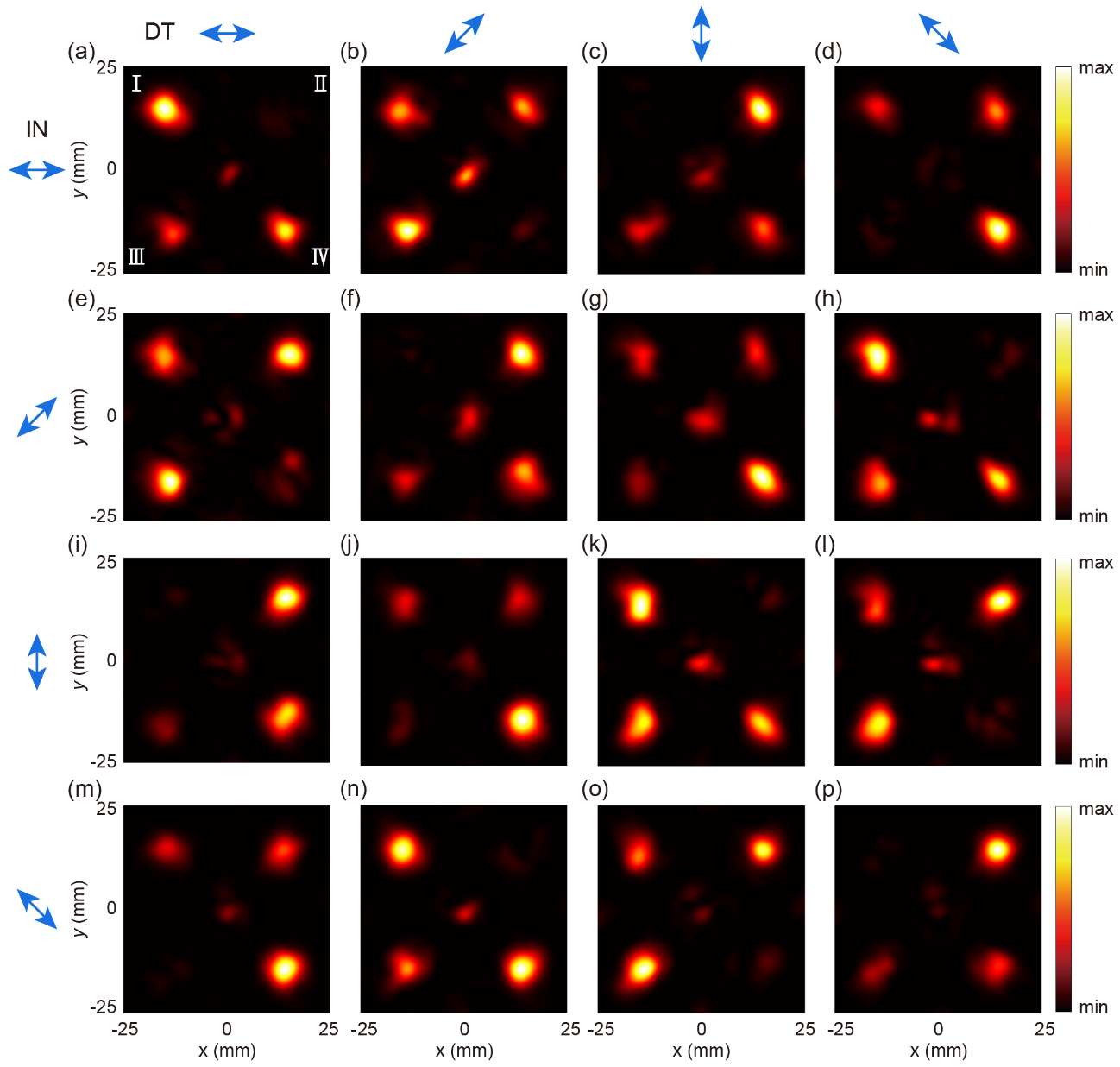


**Figure S13.** Electromagnetic simulation results of the M-4D sample, considering FP interference with a substrate thickness of 65 μm. (a-d), (e-h), (i-l), and (m-p) Electric field intensity components at 0, π/4, π/2, and 3π/4 polarization directions under LP incidence with *γ*^in^ = 0, π/4, π/2, and 3π/4, respectively, where the green arrows indicate theoretically predicted polarization direction of each beam. The blue arrows in the left column represent the incident polarization (IN), while those in the top row represent the detection polarization (DT).


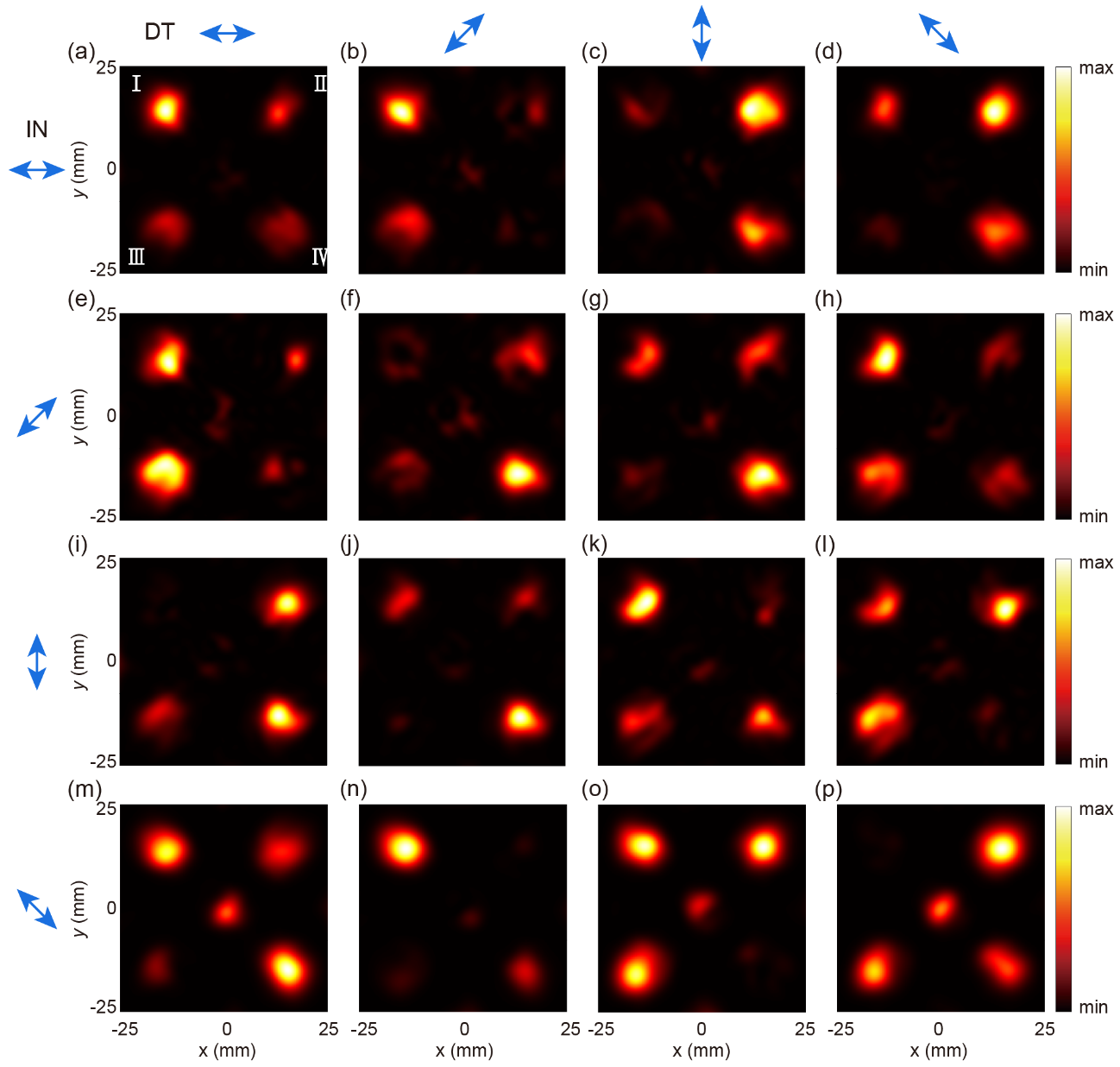


**Figure S14.** Electromagnetic simulation results of the M-4D sample, considering FP interference with a substrate thickness of 100 μm. (a-d), (e-h), (i-l), and (m-p) Electric field intensity components at 0, π/4, π/2, and 3π/4 polarization directions under LP incidence with *γ*^in^ = 0, π/4, π/2, and 3π/4, respectively, where the green arrows indicate theoretically predicted polarization direction of each beam. The blue arrows in the left column represent the incident polarization (IN), while those in the top row represent the detection polarization (DT).


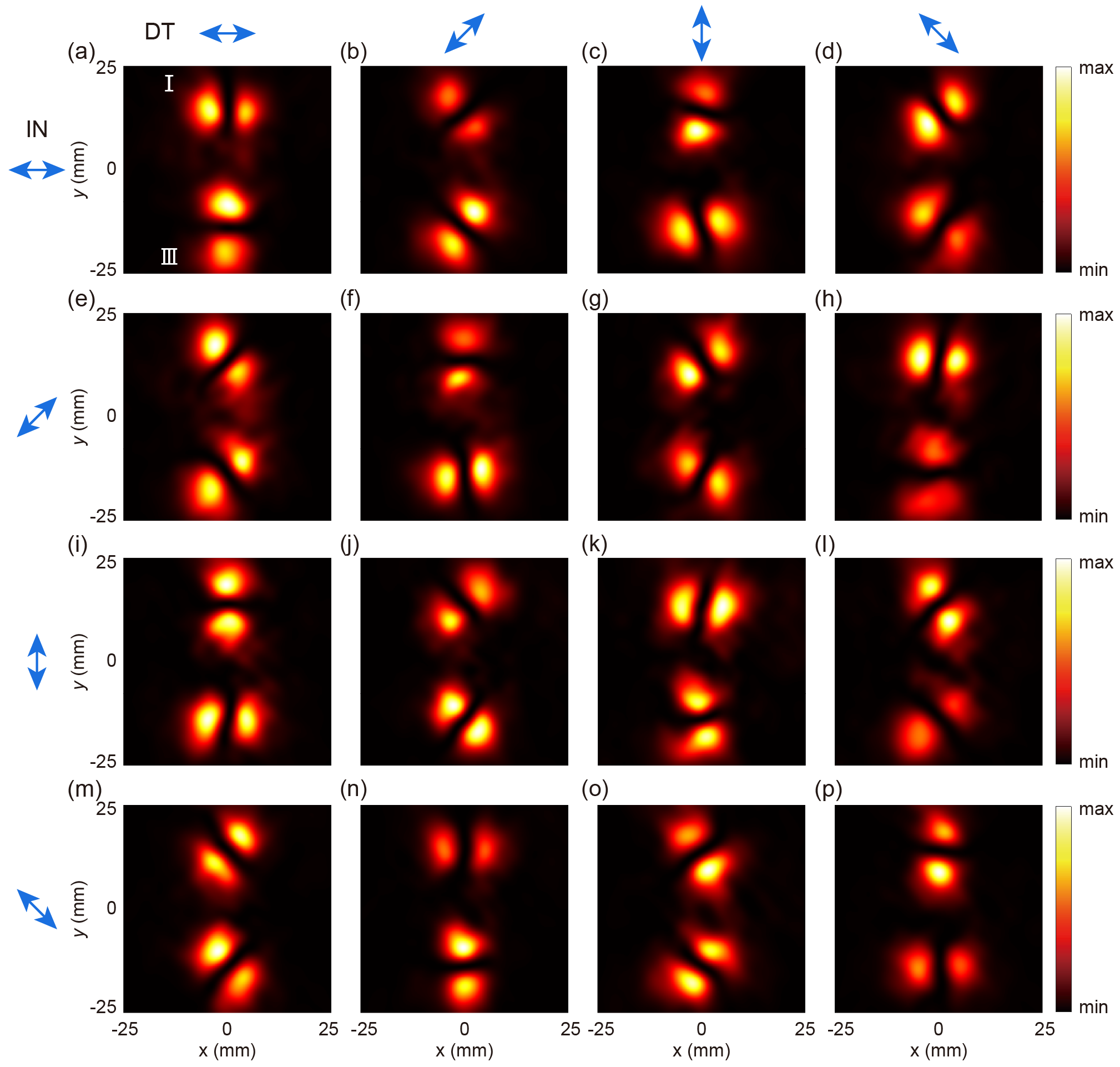


**Figure S15.** Electromagnetic simulation results of the M-2B sample, considering FP interference with a substrate thickness of 50 μm. (a-d), (e-h), (i-l), and (m-p) Electric field intensity components at 0, π/4, π/2, and 3π/4 polarization directions under LP incidence with *γ*^in^ = 0, π/4, π/2, and 3π/4, respectively, where the green arrows indicate theoretically predicted polarization direction of each beam. The blue arrows in the left column represent the incident polarization (IN), while those in the top row represent the detection polarization (DT).

**Section 11. Analysis of the impact of fabrication-related errors**

Microscopic measurements of the fabricated M-4D sample in the central region revealed that the lateral dimensions of the Si pillars were approximately reduced to 95% of the designed values. Using a white-light confocal microscope, the pillar height in the central region was measured to be approximately 190 µm. Additionally, the dielectric properties of the HR-Si wafer used in the fabrication were characterized using a THz TDS. At a frequency of 1.0 THz, the experimentally measured relative permittivity was 11.67, and the dielectric loss tangent was 0.0002. Simulations of the M-4D and M-2B devices were conducted using parameters consistent with the measured geometric and dielectric properties. The simulation results are shown in **Figure S16** and **S17**, respectively. The simulations reveal a prominent 0th-order directly transmitted beam at the center, and the average total efficiency of the four working beams under four incident polarization states for the M-4D device was 18.91%, which is lower than ideal. It is primarily attributed to fabrication errors, which caused deviations in the unit cell response from the ideal half-wave plate behavior, thereby affecting the overall device performance. As three examples, the lateral dimensions of the 32 unit structures scaled to 90%, 95%, and 110% of their original sizes to analyze the response of unit structures. **Figure S18**(a–c) illustrate the transmission amplitudes of the eigen LP components along the two principal axes *f* and *s* for the 32 unit structures in these three examples, while Figure S18(d–f) present the corresponding phase responses. It can be observed that altering the dimensions of the unit structures affects their electromagnetic responses, with some structures no longer exhibiting the ideal characteristics of a half-wave plate. As shown in Figure S18(g–i), changes in the structure dimensions result in insufficient transmission conversion to orthogonal circular polarization for some unit structures in the circular polarization basis. This leads to the degradation of the overall device performance, such as the emergence of direct zero-order transmitted beams and reduced operational efficiency. Electromagnetic simulations were conducted for six cases with modified parameters for the entire M-4D device, as summarized in **Table S1**.

1. Case C-S0 represents the original design parameters, consistent with those in the main text. The simulation results are shown in Figure 2 in the main text.
2. Cases C-S1 and C-S2 involve altering only the lateral dimensions of all Si pillars while keeping other parameters constant. The simulation results are shown in **Figure S19** and **S20**.
3. Cases C-S3 and C-S4 involve altering only the heights of all Si pillars, with other parameters unchanged. The simulation results are shown in **Figure S21** and **S22**.
4. Case C-S5 involves changing only the relative permittivity of Si while keeping all other parameters fixed. The simulation results are shown in **Figure S23**.
5. Case C-S6 corresponds to the actual fabricated sample. The simulation results are shown in Figure S16.

**Table S1.** Configuration parameters used in electromagnetic simulations and the corresponding total efficiencies of the four working beams across seven cases. Notably, Case C-S0 represents the designed M-4D configuration, with a pillar height of 200 µm and a relative permittivity of 11.9 without loss.

| Case Number | C-S0 | C-S1 | C-S2 | C-S3 | C-S4 | C-S5 | C-S6 |
| --- | --- | --- | --- | --- | --- | --- | --- |
| Change in Lateral Size of Pillars | / | Scale to 90% | Scale to 110% | / | / | / | Scale to 95% |
| Change in Height of Pillars | / | / | / | 180 μm | 220 μm | / | 190 μm |
| Relative Permittivity | / | / | / | / | / | 11.305 | 11.67 |
| Dielectric Loss Tangent | / | / | / | / | / | / | 0.0002 |
| Efficiency | 22.72% | 19.96% | 18.23% | 18.8% | 22.62% | 20.56% | 18.91% |

The simulation results indicate that changes in the lateral dimensions of the unit structures lead to prominent zero-order direct transmission beams, significantly impacting device performance. In comparison, variations in the heights of the unit structures appear to have a less pronounced effect. The relative permittivity of Si also influences the device to some extent. The efficiencies of the four beams under each scenario are summarized in Table S1, showing a reduction in efficiency across all cases. The observed performance degradation is fundamentally attributed to changes in the parameters of the unit structures, which alter the transmission responses of the eigen LP components along the two principal axes *f* and *s*. Although parameter variations may lead to some performance degradation, the overall performance of output four beams remains consistent with theoretical expectations, demonstrating a degree of robustness. Despite certain deviations in the fabricated device, its overall performance still aligns well with the theoretical predictions. While considering the FP interference caused by the substrate, additional electromagnetic simulations were performed for the M-4D and M-2B devices, assuming a substrate thickness of 50 µm and using the same parameters as the measurements. The results are shown in **Figure S24** and **S25**, respectively. These simulations reveal that, in addition to fabrication errors, the unfavorable experimental outcomes compared to simulations are significantly influenced by the detrimental effects of FP interference due to the thick substrate.


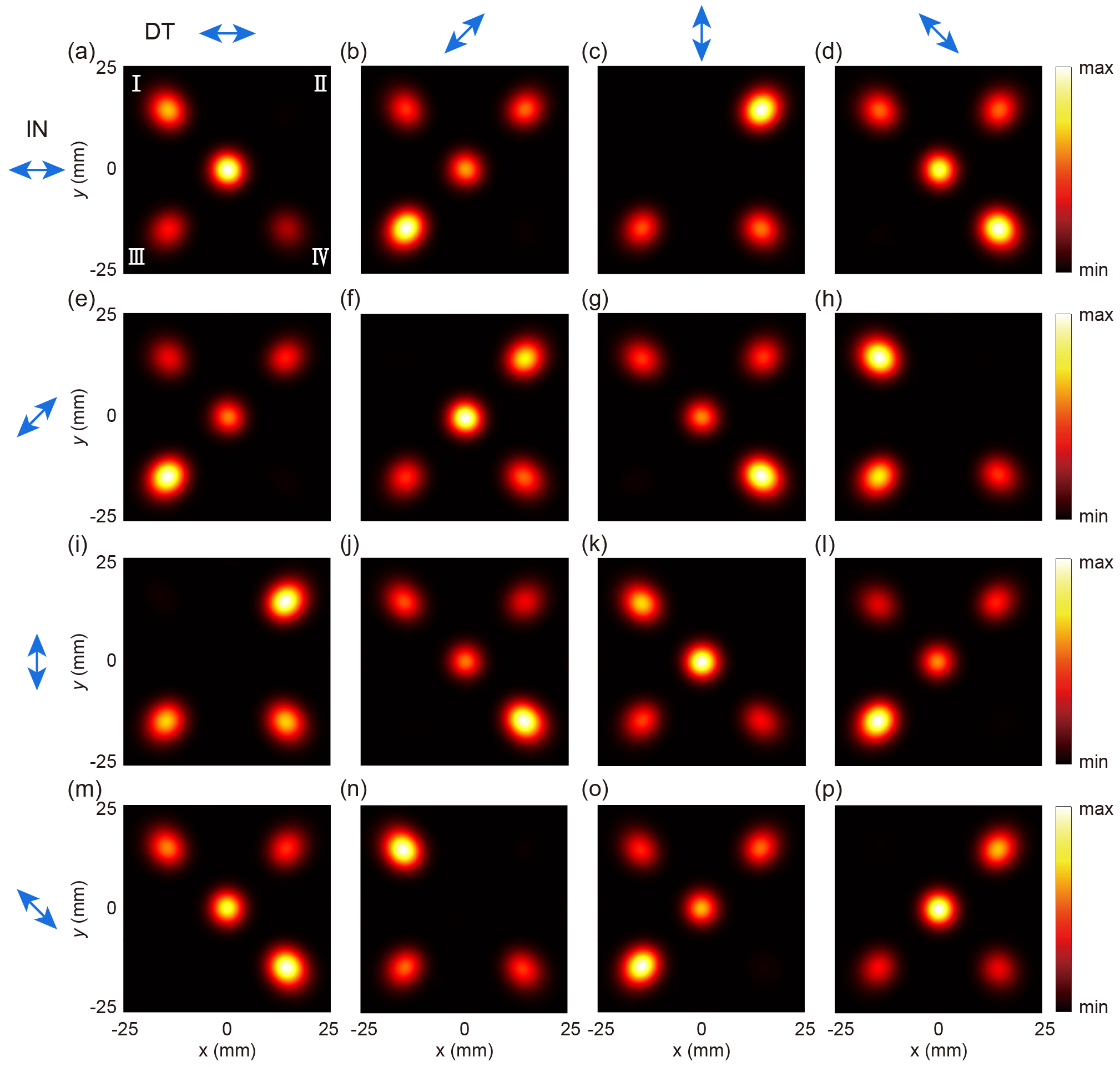


**Figure S16.** Simulation results of the meta-device M-4D with all Si pillars' lateral dimensions scaled to 95%, height set to 190 μm, relative permittivity set to 11.67 and dielectric loss tangent set to 0.0002 (Case C-S6). (a-d), (e-h), (i-l), and (m-p) Electric field intensity components at 0, π/4, π/2, and 3π/4 polarization directions under LP incidence with *γ*^in^ = 0, π/4, π/2, and 3π/4, respectively, where the green arrows indicate theoretically predicted polarization direction of each beam. The blue arrows in the left column represent the incident polarization (IN), while those in the top row represent the detection polarization (DT).


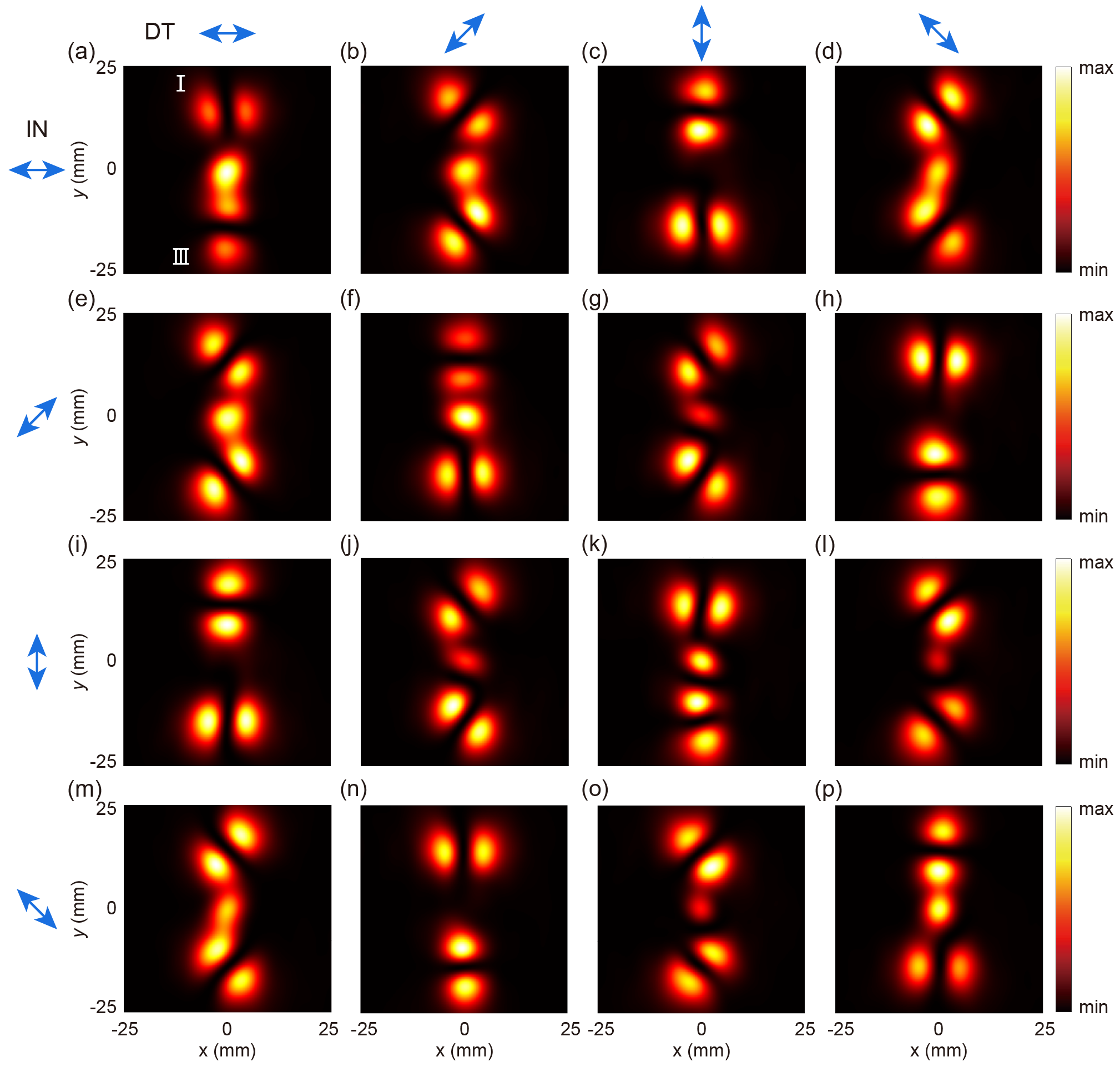


**Figure S17.** Simulation results of the meta-device M-2B with all Si pillars' lateral dimensions scaled to 95%, height set to 190 μm, relative permittivity set to 11.67 and dielectric loss tangent set to 0.0002. (a-d), (e-h), (i-l), and (m-p) Electric field intensity components at 0, π/4, π/2, and 3π/4 polarization directions under LP incidence with *γ*^in^ = 0, π/4, π/2, and 3π/4, respectively, where the green arrows indicate theoretically predicted polarization direction of each beam. The blue arrows in the left column represent the incident polarization (IN), while those in the top row represent the detection polarization (DT).


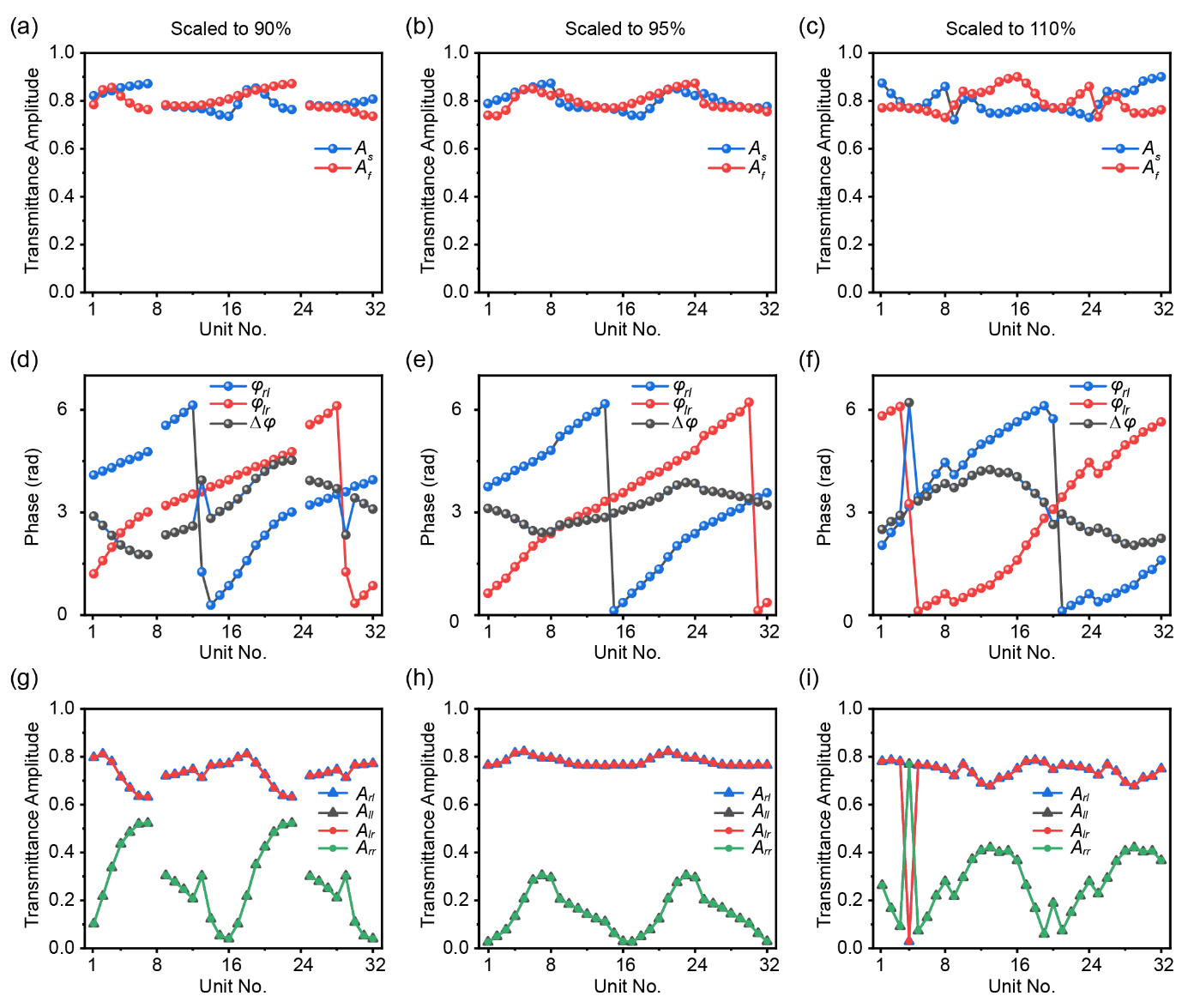


**Figure S18.** Electromagnetic responses of the 32 unit structures at 1.0 THz, with lateral dimensions scaled to 90%, 95%, and 110% of the original designed size. (a), (d), (g): Eigen transmission amplitude profiles, eigen phase shift profiles, and CP transmission amplitude profiles, respectively, at a scaling ratio of 90%. (b), (e), (h): Eigen transmission amplitude profiles, eigen phase shift profiles, and CP transmission amplitude profiles, respectively, at a scaling ratio of 95%. Note that structures No. 8 (29.25 µm, 75.6 µm) and No. 24 (75.6 µm, 29.25 µm) are outside the database's scanning range, so their data are not listed. (c), (f), (i): Eigen transmission amplitude profiles, eigen phase shift profiles, and CP transmission amplitude profiles, respectively, at a scaling ratio of 110%.


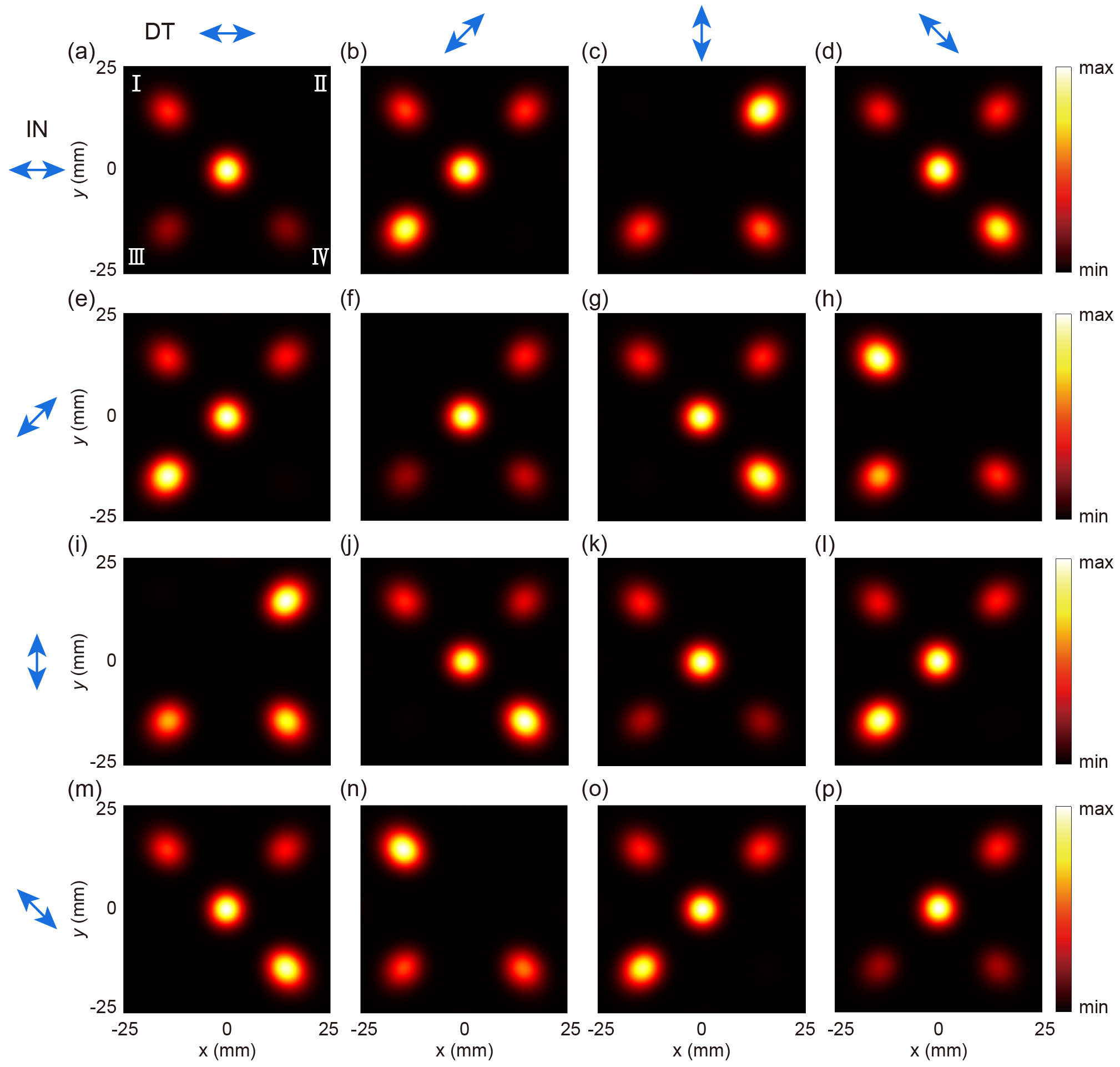


**Figure S19.** Simulation results of the meta-device M-4D with all Si pillars' lateral dimensions scaled to 90% (Case C-S1). (a-d), (e-h), (i-l), and (m-p) Electric field intensity components at 0, π/4, π/2, and 3π/4 polarization directions under LP incidence with *γ*^in^ = 0, π/4, π/2, and 3π/4, respectively. The blue arrows in the left column represent the incident polarization (IN), while those in the top row represent the detection polarization (DT).


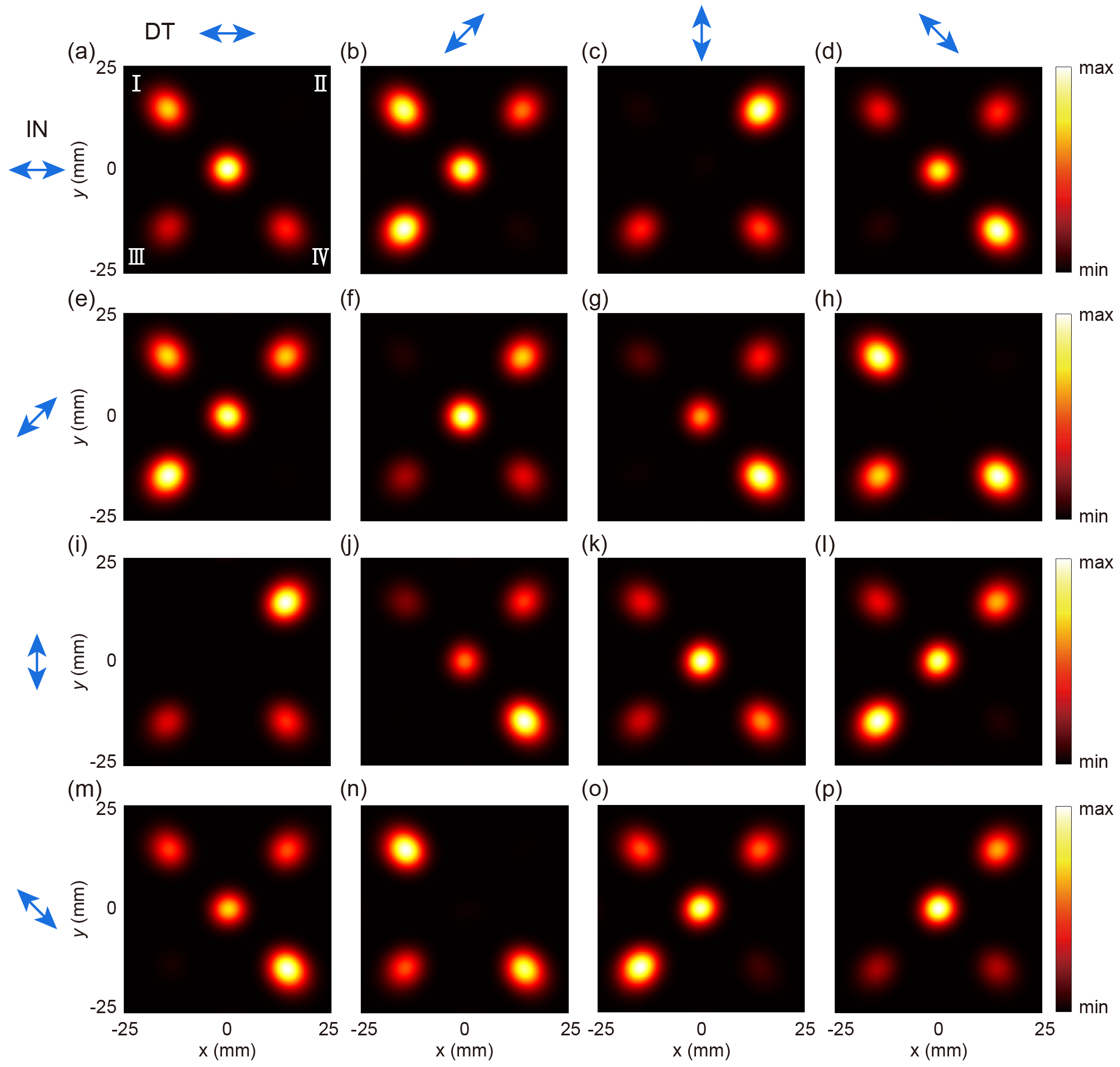


**Figure S20.** Simulation results of the meta-device M-4D with all Si pillars' lateral dimensions scaled to 110% (Case C-S2). (a-d), (e-h), (i-l), and (m-p) Electric field intensity components at 0, π/4, π/2, and 3π/4 polarization directions under LP incidence with *γ*^in^ = 0, π/4, π/2, and 3π/4, respectively. The blue arrows in the left column represent the incident polarization (IN), while those in the top row represent the detection polarization (DT).


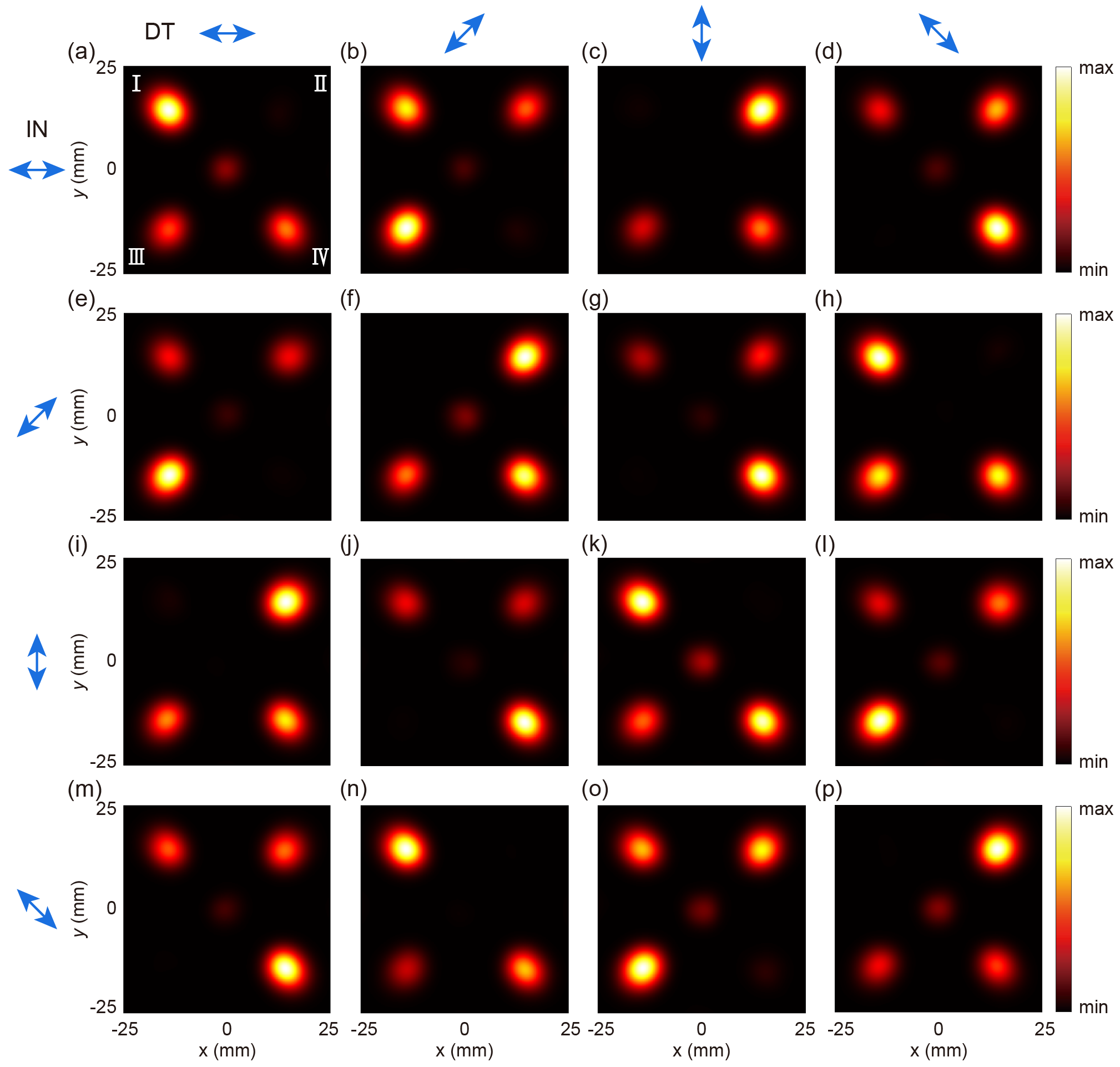


**Figure S21.** Simulation results of the meta-device M-4D with all Si pillars' height set to 180 μm (Case C-S3). (a-d), (e-h), (i-l), and (m-p) Electric field intensity components at 0, π/4, π/2, and 3π/4 polarization directions under LP incidence with *γ*^in^ = 0, π/4, π/2, and 3π/4, respectively. The blue arrows in the left column represent the incident polarization (IN), while those in the top row represent the detection polarization (DT).


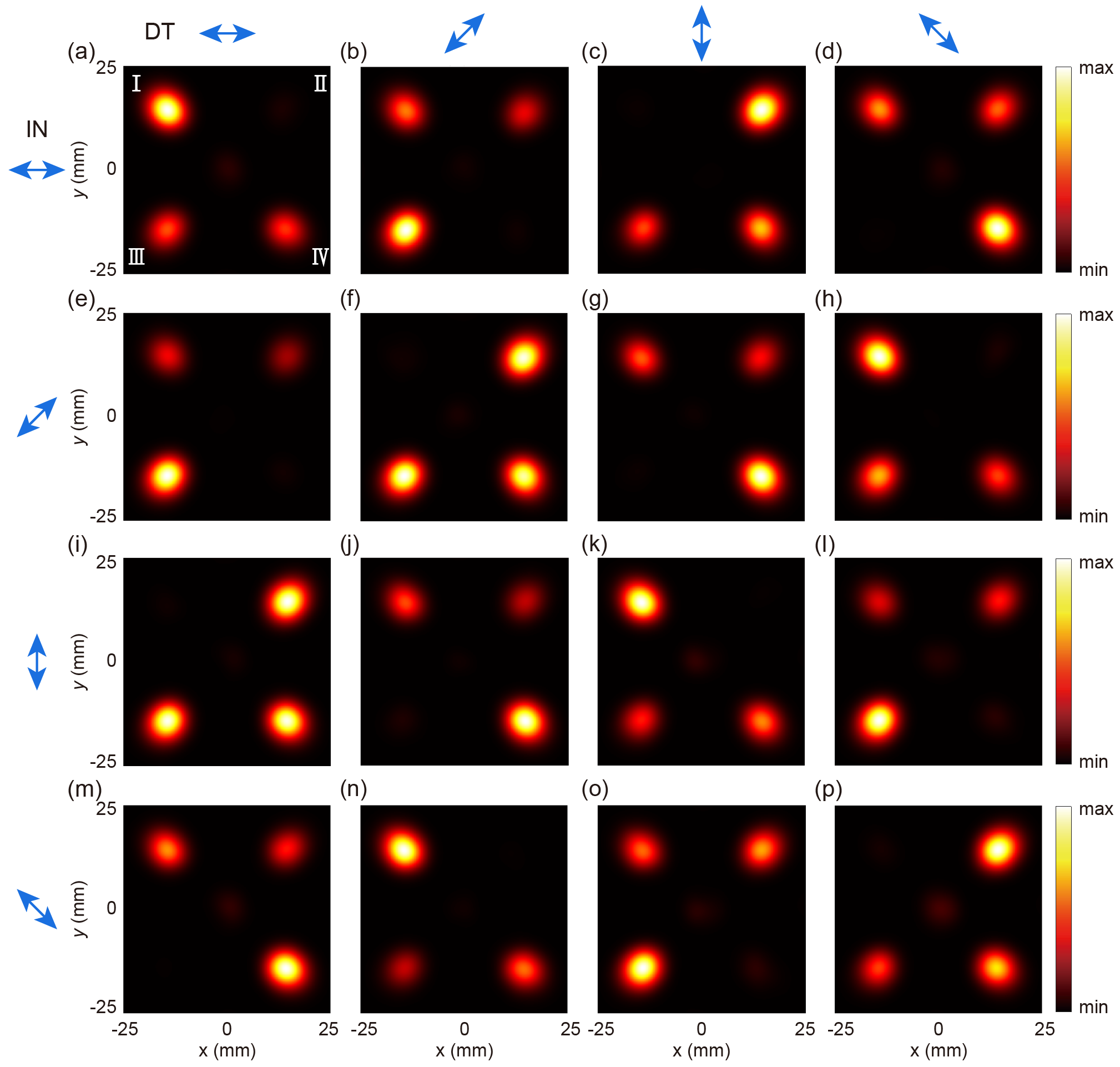


**Figure S22.** Simulation results of the meta-device M-4D with all Si pillars' height set to 220 μm (Case C-S4). (a-d), (e-h), (i-l), and (m-p) Electric field intensity components at 0, π/4, π/2, and 3π/4 polarization directions under LP incidence with *γ*^in^ = 0, π/4, π/2, and 3π/4, respectively. The blue arrows in the left column represent the incident polarization (IN), while those in the top row represent the detection polarization (DT).


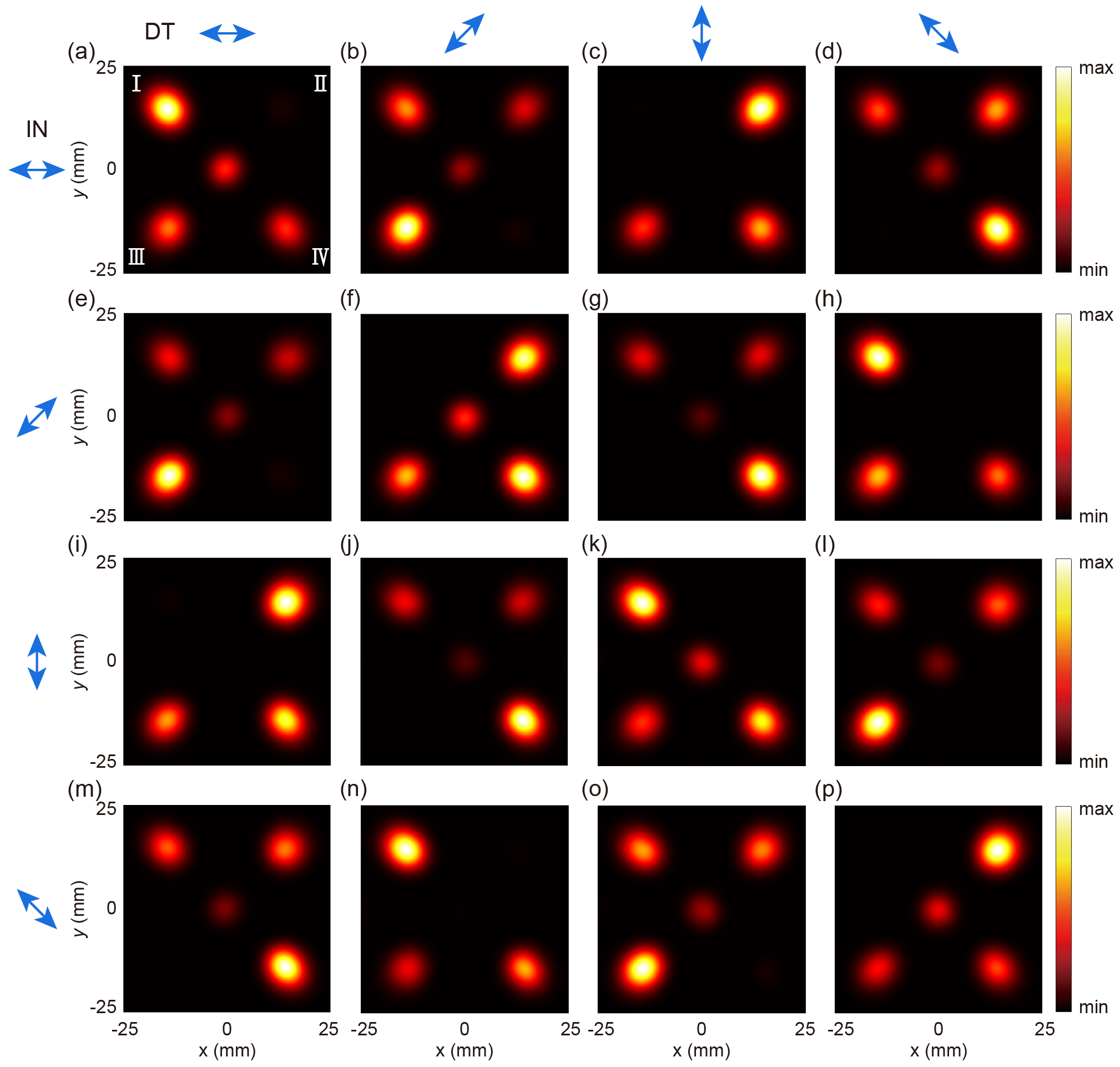


**Figure S23.** Simulation results of the meta-device M-4D with all Si pillars' relative permittivity set to 11.305 (Case C-S5). (a-d), (e-h), (i-l), and (m-p) Electric field intensity components at 0, π/4, π/2, and 3π/4 polarization directions under LP incidence with *γ*^in^ = 0, π/4, π/2, and 3π/4, respectively. The blue arrows in the left column represent the incident polarization (IN), while those in the top row represent the detection polarization (DT).

**
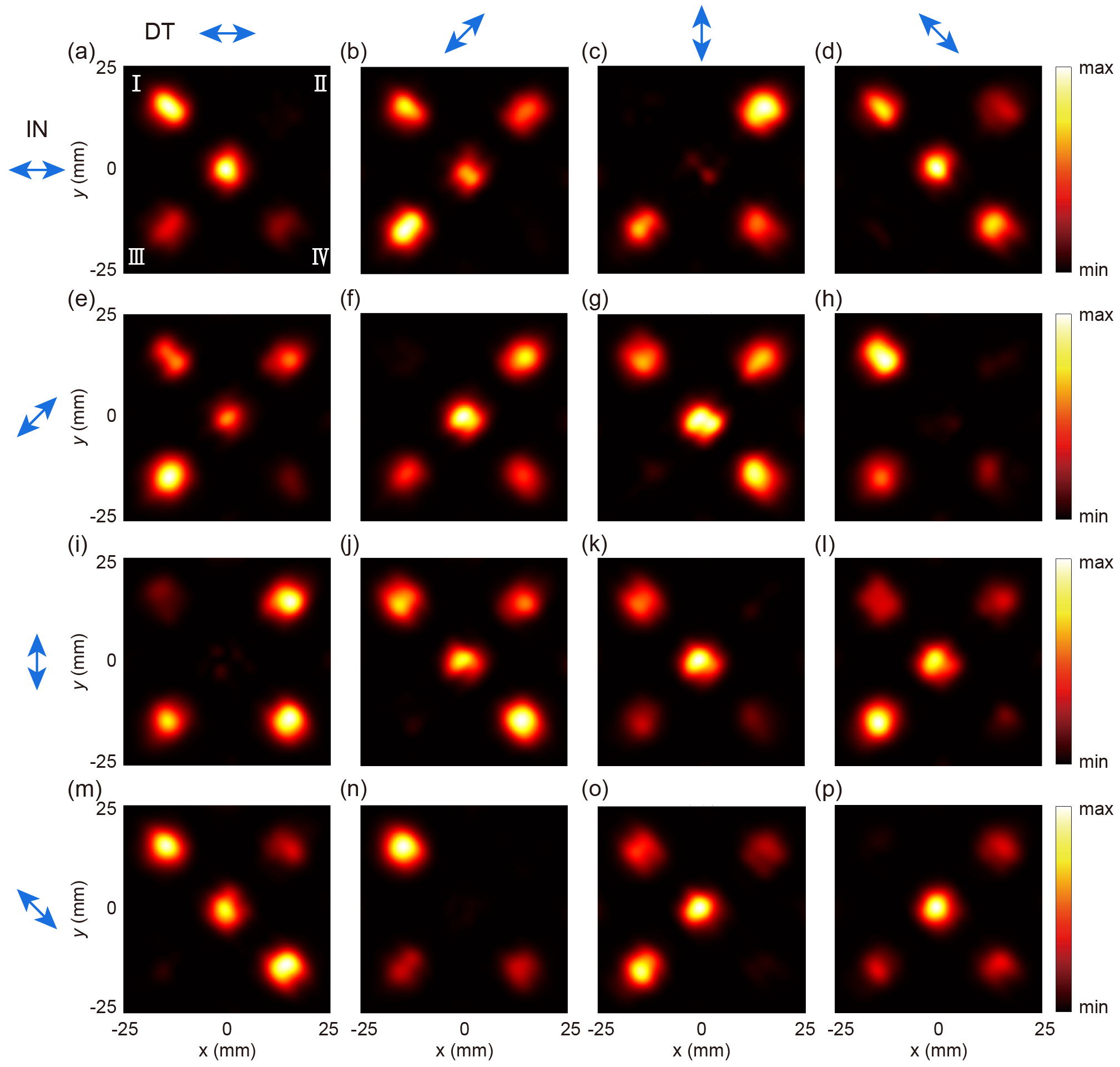
**

**Figure S24.** Simulation results of the meta-device M-4D with all Si pillars' lateral dimensions scaled to 95%, height set to 190 μm, relative permittivity set to 11.67, dielectric loss tangent set to 0.0002 and FP conference substrate thickness set as 50 μm. (a-d), (e-h), (i-l), and (m-p) Electric field intensity components at 0, π/4, π/2, and 3π/4 polarization directions under LP incidence with *γ*^in^ = 0, π/4, π/2, and 3π/4, respectively, where the green arrows indicate theoretically predicted polarization direction of each beam. The blue arrows in the left column represent the incident polarization (IN), while those in the top row represent the detection polarization (DT).


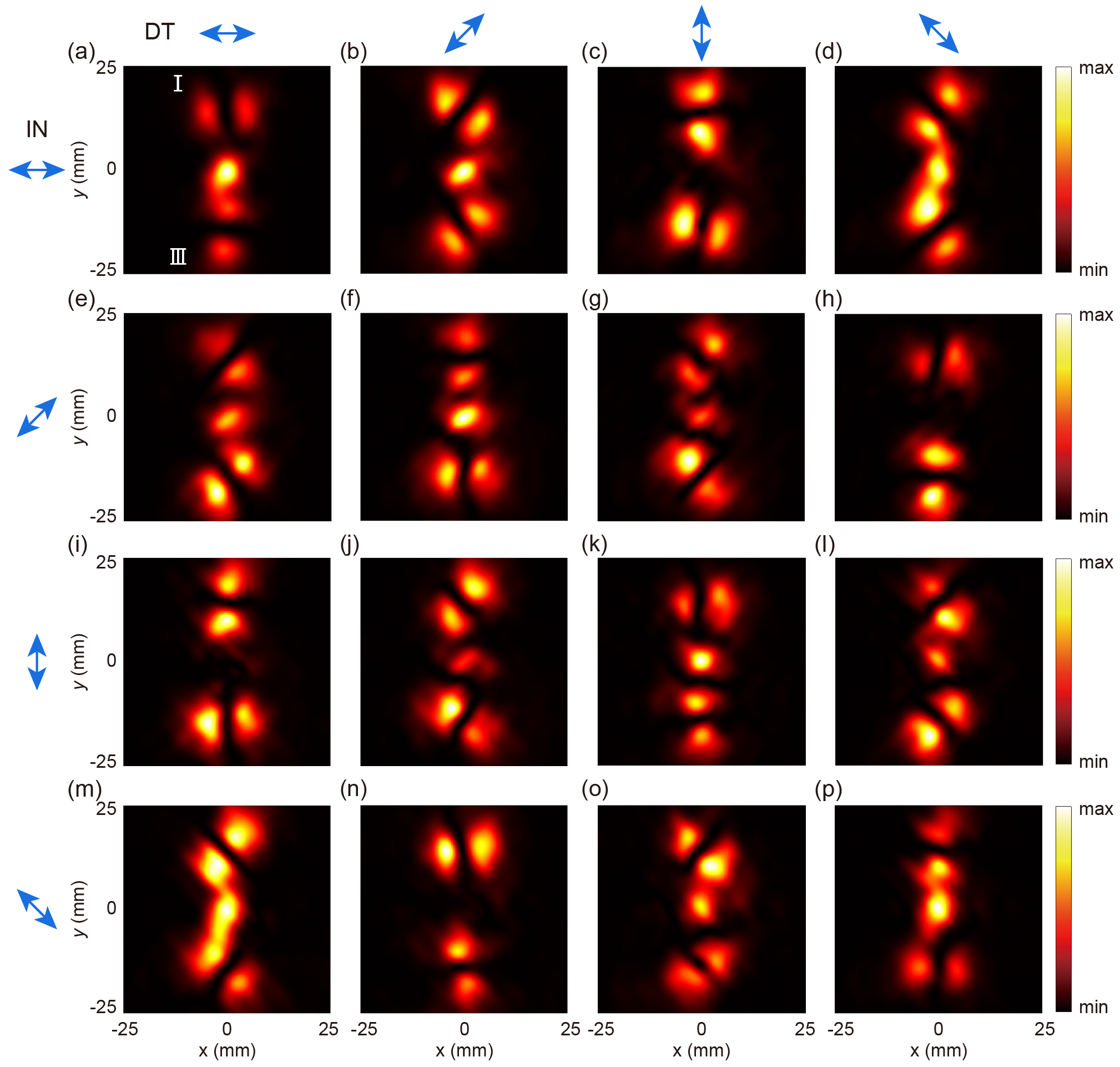


**Figure S25.** Simulation results of the meta-device M-2B with all Si pillars' lateral dimensions scaled to 95%, height set to 190 μm, relative permittivity set to 11.67, dielectric loss tangent set to 0.0002 and FP conference substrate thickness set as 50 μm. (a-d), (e-h), (i-l), and (m-p) Electric field intensity components at 0, π/4, π/2, and 3π/4 polarization directions under LP incidence with *γ*^in^ = 0, π/4, π/2, and 3π/4, respectively, where the green arrows indicate theoretically predicted polarization direction of each beam. The blue arrows in the left column represent the incident polarization (IN), while those in the top row represent the detection polarization (DT).

**Section 12. Analysis of High-Order Diffraction and Efficiency**

Based on HFSS simulations of the M-4D device, the total electric field intensity distribution on a hemispherical surface with a radius of *r* = 55 mm was calculated, as shown in **Figure S26**. In the figure, latitude lines represent polar angles, while longitude lines denote azimuthal angles. It can be observed that, in addition to the four primary working beams corresponding to the (±1, ±1) diffraction orders, there are noticeable high-order diffraction components. These higher-order diffractions reduce the overall efficiency of the device to some extent. To further investigate this issue, purely numerical calculations were performed using the Rayleigh-Sommerfeld diffraction formula for ideal phase distributions with varying phase gradient orders (*N*) and periods (*P*). Configurations C1–C3 represent cases where *N* = 8 is fixed while *P* is varied, and configurations C3–C6 represent cases where the super-period *NP* is fixed while both *N* and *P* are simultaneously adjusted. Configuration C3 corresponds to the meta-device M-4D design described in the main text. Detailed parameter settings are provided in **Table S2**. **Figure S27** presents the electric field intensity distributions on the hemispherical surface for each configuration. The results for C3 show that the purely numerical calculations closely match the HFSS electromagnetic simulation results. The total efficiency of the four working beams is calculated to be 23.42%, while the HFSS simulated efficiency is 22.65%, demonstrating strong consistency and validating the simulation results. Compared to C3, configurations C1, C5, and C6 exhibit significantly reduced high-order diffraction orders, whereas configurations C2 and C3 show more prominent high-order diffraction orders. The total efficiency of the four working beams remains approximately 23% across all configurations, as summarized in Table S2.

**Table S2.** Configuration parameters and corresponding total efficiencies of the four working beams for the six cases. Notably, case C3 represents the designed M-4D configuration.

| Case Number | C1 | C2 | C3 | C4 | C5 | C6 |
| --- | --- | --- | --- | --- | --- | --- |
| N: Number of Units | 8 | 8 | 8 | 6 | 12 | 16 |
| P: Period of Unit [μm] | 112.5 | 187.5 | 150 | 200 | 100 | 75 |
| NP: Super Period [μm] | 900 | 1500 | 1200 | 1200 | 1200 | 1200 |
| Efficiency | 22.12% | 24.00% | 23.42% | 23.72% | 23.41% | 23.40% |

Further analysis of far-field diffraction using the Fourier method was conducted for configurations C1 and C3. **Figure S28**(a) and (b) illustrate the relationships between spatial frequencies *k_x_*​, *k_y_*_​_, and *k_z_*^2^​, given by the expression *k_z_*^2^​ = *k*^2^ − *k_x_*^2^ − *k_y_*^2^ ​, where *k* = 1/*λ*. Figure S28(c) and (d) show the intensity distributions of far-field diffraction components in the *k_x_*–*k_y_* plane for C1 and C3, respectively. It is observed that only within the yellow circles does *k_z_*^2^ > 0, indicating real *k_z_*, which corresponds to propagating waves. Outside the yellow circles, *k_z_*^2^ < 0, resulting in complex *k_z_*​, which corresponds to evanescent waves. These waves are confined to the near field and cannot propagate to the far field. While these high-order evanescent waves do not contribute to the far field, they consume input energy and are a primary factor limiting device efficiency. For configuration C3, the number of far-field propagating diffraction orders is 12, consistent with Figure S26 and S27(c), with the total efficiency of the four working beams (±1, ±1 orders) reaching 25%. For configuration C1, only 4 diffraction orders propagate to the far field, consistent with Figure S27(a), and the total efficiency of the four working beams is also 25%. **Figure S29** summarizes the total efficiency of the four working beams across the six configurations calculated using the Rayleigh-Sommerfeld diffraction formula and Fourier analysis. The results from the two methods are in excellent agreement, demonstrating that adjusting *N* and *P* cannot significantly enhance device efficiency due to the inherent limitations of the phase design method, with a theoretical upper efficiency limit of 25%. However, appropriately tuning *N* and *P* can effectively suppress stray high-order diffraction orders (e.g., in C1, C5, and C6), resulting in purer working beams in the far field.


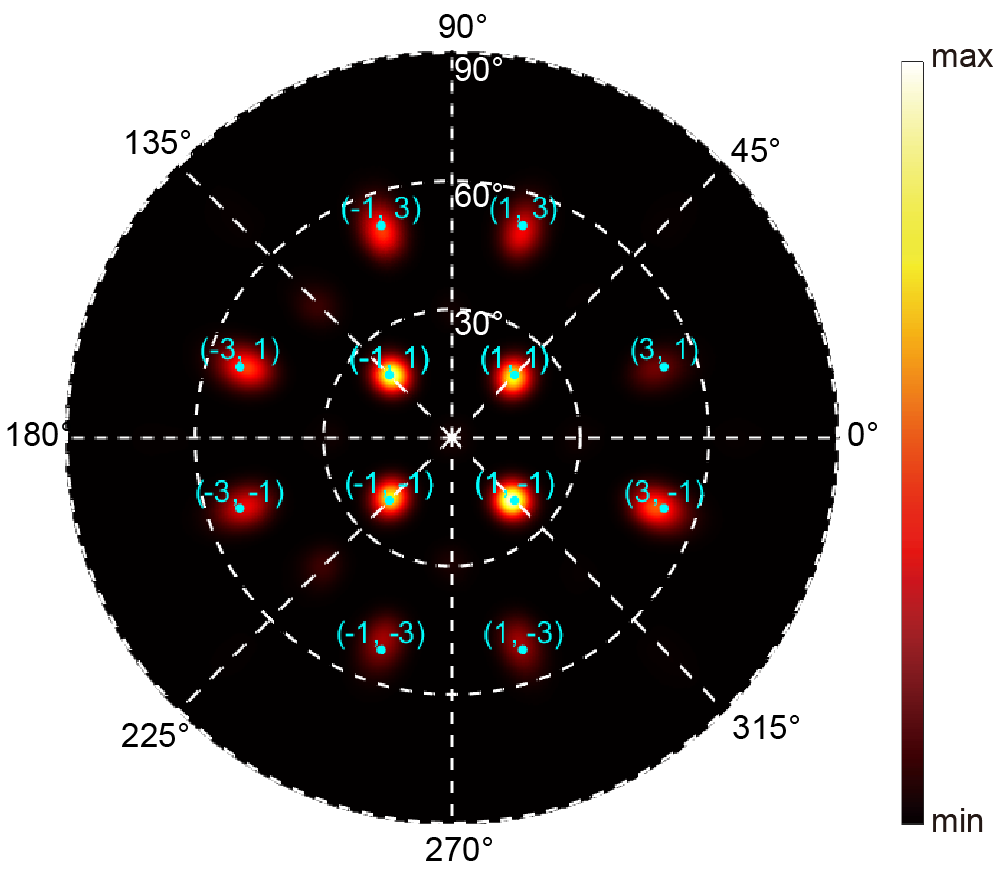


**Figure S26.** Total electric field intensity distribution on a hemispherical surface with a radius of *r* = 55 mm, calculated using HFSS simulation data for the M-4D device. Latitude lines represent polar angles, while longitude lines denote azimuthal angles.


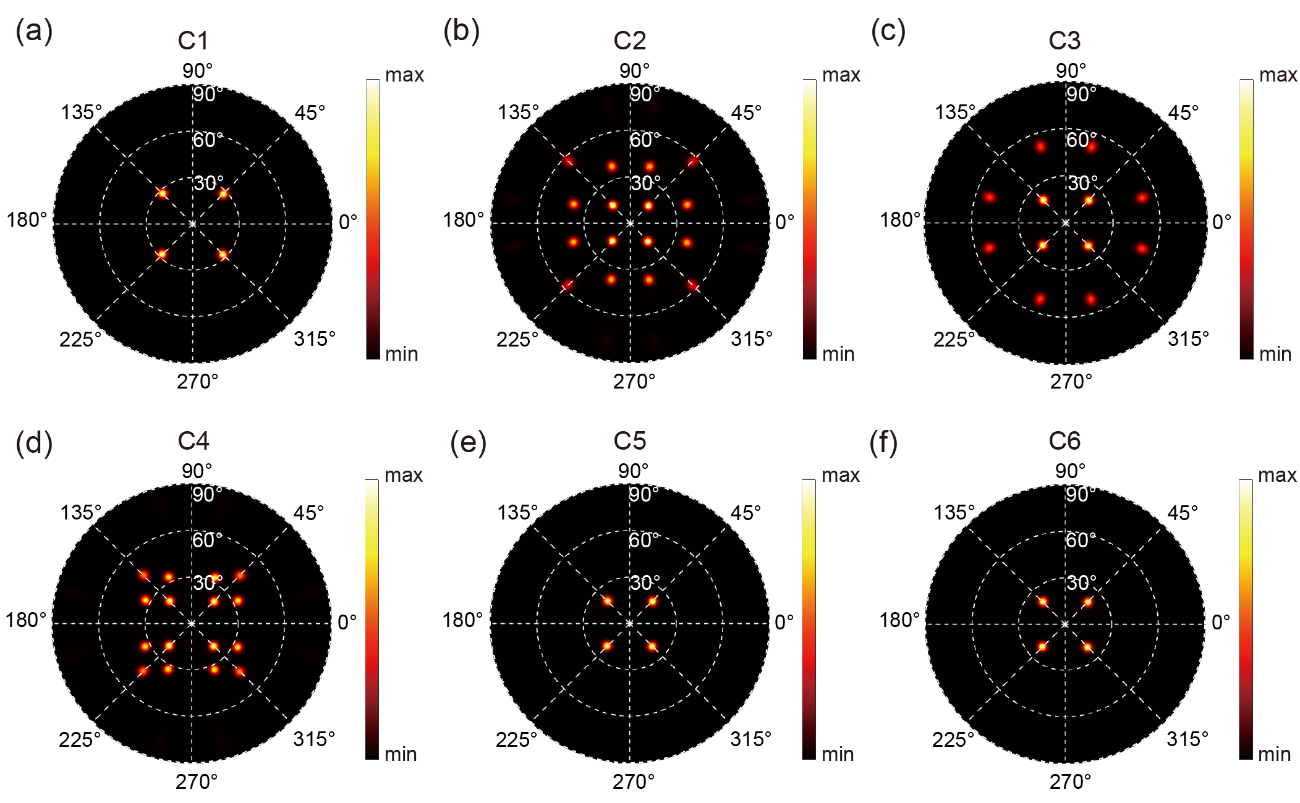


**Figure S27.** Total electric field intensity distributions on a hemispherical surface with a radius of r = 55 mm, obtained using the Rayleigh-Sommerfeld diffraction analysis method for six cases. Panels (a–f) correspond to Case 1 through Case 6, respectively.


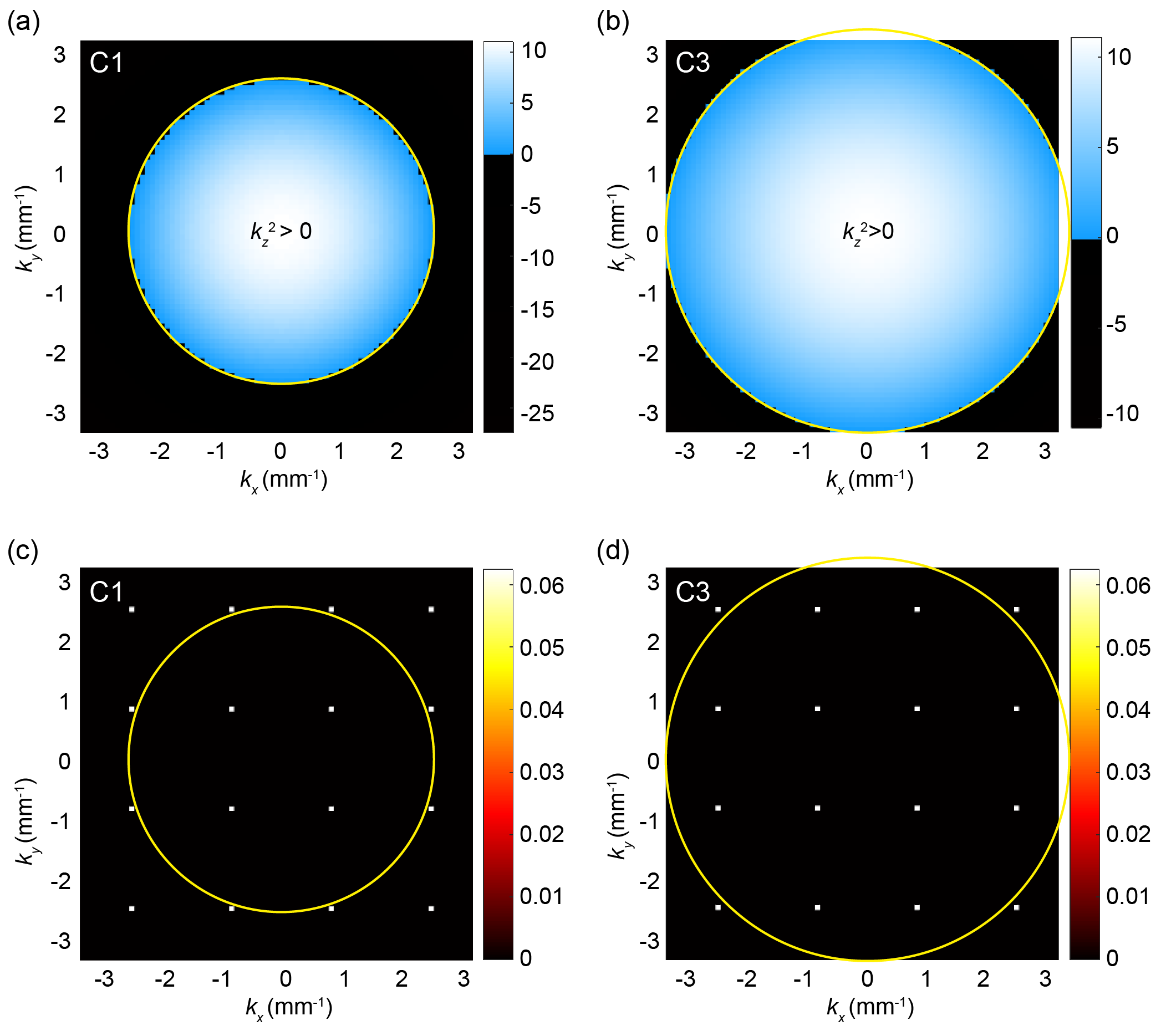


**Figure S28.** Far-field diffraction analysis results for cases C1 and C3 using the Fourier analysis method. (a) and (b) depict the relationships among spatial frequencies *k_x_* ​, *k_y_*​, and *k_z_*^2^ for cases C1 and C3, respectively. (c) and (d) show the far-field diffraction intensity distributions in the spatial frequency coordinates *k_x_* and *k_y_*_​_ for cases C1 and C3, respectively.


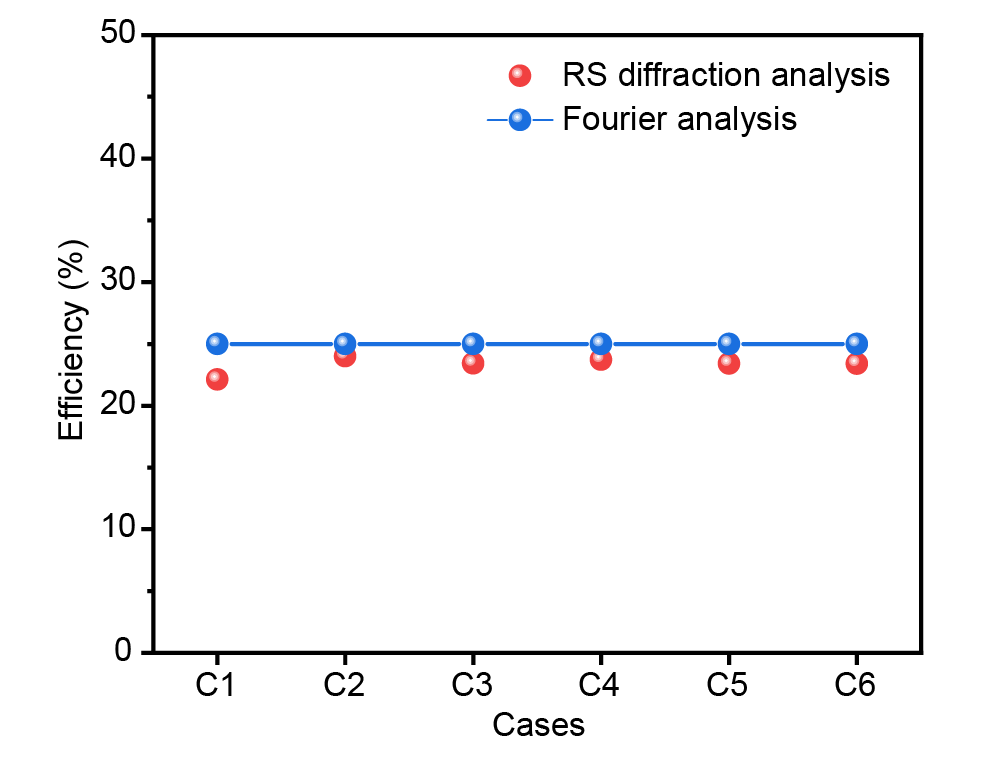


**Figure S29.** The total efficiency of the four working beams calculated using the Rayleigh-Sommerfeld diffraction analysis method and the Fourier analysis method in six cases.

Reference

1. Y. H. Xu, H. F. Zhang, Q. Li, et al., "Generation of terahertz vector beams using dielectric metasurfaces via spin-decoupled phase control," Nanophotonics 9, 3393-3402 (2020).
2. H. Minamide, S. Hayashi, K. Nawata, et al., "Kilowatt-peak terahertz-wave generation and sub-femtojoule terahertz-wave pulse detection based on nonlinear optical wavelength-conversion at room temperature," Journal of Infrared Millimeter and Terahertz Waves 35, 25-37 (2014).
